# Correction: Cardiovascular risk in aging adults with double deficits in social support: A gender-sensitive, cross-sectional analysis of the CLSA cohort

**DOI:** 10.1371/journal.pone.0337174

**Published:** 2025-11-18

**Authors:** Annalijn I. Conklin, Abdollah Safari, Gerry Veenstra, Nadia A. Khan

The Disclaimer statement is omitted from the article. It can be viewed below.

The opinions expressed in this manuscript are the author’s own and do not reflect the views of the Canadian Longitudinal Study on Aging.
