# Supplementary material for: Cardiovascular risk in aging adults with double deficits in social support: A gender-sensitive, cross-sectional analysis of the CLSA cohort
Source: PLoS One. 2025 Jun 20;20(6):e0309634. doi: 10.1371/journal.pone.0309634 (PMC12180724; doi:10.1371/journal.pone.0309634)
Supplement: S1 File — (DOCX) [file pone.0309634.s001.docx]

**Cardiovascular risk in aging adults with double deficits in social support: a gender-sensitive, cross-sectional analysis of the CLSA cohort**

**Supplementary Materials and Methods**

**Study design and population**

The Canadian Longitudinal Study on Aging (CLSA) is a population-based cohort of middle-age and older adults (45-85 years at baseline, 2011-2015) with the goal of understanding the factors that affect health and wellness in older people. The CLSA used a combination of sampling methods to recruit participants and collect data. These methods include random digit dialing (to recruit participants by calling landline telephones), recruitment from health care registries (to recruit participants from provincial health care systems), and over-sampling of certain areas (to ensure that groups with lower socioeconomic status and less education are represented). The CLSA Comprehensive cohort included 30,098 participants randomly selected from within 25-50 km of 11 Data Collection Sites (DCS) in seven provinces. These participants provided self-reported information on health and wellbeing through in-person interviews using a structured questionnaire and were clinically measured through physical assessment.

**Four types of social support**

The CLSA DCS structured questionnaire includes a number of modules that are copyright protected; the Neuropsychological Questionnaire included a module on Social Support Availability (SSA) to assess the multidimensional concept of functional social support using the 19-item Medical Outcomes Study (MOS) Social Support Survey. The MOS survey is considered to have higher reliability as multiple studies consistently report excellent internal consistency (all Cronbach’s alpha values are commonly above 0.90); this indicates that there is strong internal consistency among the 19 MOS items and that the MOS measures a stable and consistent construct across different samples. Not only is there good test-retest reliability scores across various versions of the scale, but the reliability of each subscale also remains high. The CLSA’s module on Social Support Availability (SSA) measures CLSA participants’ perception that one is in receipt of material aid or behavioral assistant; expressions of love and affection, positive affect, empathetic understanding and encouragement of expressions of feelings; positive companionship; or the offering of advice information, guidance and feedback. The CLSA provides variables for each MOS question item as well as five derived variables in the Social Support Availability module. Whereas one of the MOS subscales combines emotional with informational support, we considered the expression of concern, affection, caring and providing comfort and encouragement to be distinct from advice and guidance. Thus, for this study, we used the specific CLSA questions from the SSA module to differentiate the four specific types of social support that an individual may experience. Based on theory from the literature and confirmatory factor analysis, we classified each SSA question in the CLSA questionnaire according to: *emotional* or appraisal support (listening support, comfort, affection and security), *informational* support (advice and guidance), *belonging* or companionship support (socializing or sharing common interests, being valued/social reinforcement), and *tangible* or instrumental support (concrete assistance such as providing transportation or financial assistance). S1 Table gives each support type corresponding to the specific CLSA question items and scoring approach.

**S1 Table. Overview of the specific social support types, CLSA questions, response options and scores used in this study**

| Specific type of social support | Question in the CLSA SSA module about how often each kind of support was available if needed | Response values for scoring |
| --- | --- | --- |
| **Emotional support**  (score: 7-35 points) | Q2. Someone you can count on to listen to you when you need to talk | 1 = none of the time; 2 = a little of the time; 3 = some of the time; 4 = most of the time; 5 = all of the time |
|  | Q5. Someone who shows you love and affection | 1 = none of the time; 2 = a little of the time; 3 = some of the time; 4 = most of the time; 5 = all of the time |
|  | Q8. Someone to confide in or talk to about yourself or your problems | 1 = none of the time; 2 = a little of the time; 3 = some of the time; 4 = most of the time; 5 = all of the time |
|  | Q9. Someone who hugs you | 1 = none of the time; 2 = a little of the time; 3 = some of the time; 4 = most of the time; 5 = all of the time |
|  | Q15 Someone to share our most private worries and fears with | 1 = none of the time; 2 = a little of the time; 3 = some of the time; 4 = most of the time; 5 = all of the time |
|  | Q18. Someone who understands your problems | 1 = none of the time; 2 = a little of the time; 3 = some of the time; 4 = most of the time; 5 = all of the time |
|  | Q19. Someone to love you and make you feel wanted | 1 = none of the time; 2 = a little of the time; 3 = some of the time; 4 = most of the time; 5 = all of the time |
| **Informational support**  (score: 4-20 points) | Q3. Someone to give you advice about a crisis | 1 = none of the time; 2 = a little of the time; 3 = some of the time; 4 = most of the time; 5 = all of the time |
|  | Q7. Someone to give you information in order to help you | 1 = none of the time; 2 = a little of the time; 3 = some of the time; 4 = most of the time; 5 = all of the time |
|  | Q16. Someone to turn to for suggestions about how to deal with a personal problem | 1 = none of the time; 2 = a little of the time; 3 = some of the time; 4 = most of the time; 5 = all of the time |
|  | Q12. Someone whose advice you really want | 1 = none of the time; 2 = a little of the time; 3 = some of the time; 4 = most of the time; 5 = all of the time |
| **Tangible support**  (score: 4-20 points) | Q1. Someone to help you if you were confined to bed | 1 = none of the time; 2 = a little of the time; 3 = some of the time; 4 = most of the time; 5 = all of the time |
|  | Q4. Someone to take you to the doctor if needed | 1 = none of the time; 2 = a little of the time; 3 = some of the time; 4 = most of the time; 5 = all of the time |
|  | Q11. Someone to prepare your meals if you were unable to do it yourself | 1 = none of the time; 2 = a little of the time; 3 = some of the time; 4 = most of the time; 5 = all of the time |
|  | Q14. Someone to help with daily chores if you were sick | 1 = none of the time; 2 = a little of the time; 3 = some of the time; 4 = most of the time; 5 = all of the time |
| **Belonging support**  (score: 4-20 points) | Q6. Someone to have a good time with? | 1 = none of the time; 2 = a little of the time; 3 = some of the time; 4 = most of the time; 5 = all of the time |
|  | Q10. Someone to get together with for relaxation | 1 = none of the time; 2 = a little of the time; 3 = some of the time; 4 = most of the time; 5 = all of the time |
|  | Q13. Someone to do things with to help you get your mind off things | 1 = none of the time; 2 = a little of the time; 3 = some of the time; 4 = most of the time; 5 = all of the time |
|  | Q17. Someone to do something enjoyable with | 1 = none of the time; 2 = a little of the time; 3 = some of the time; 4 = most of the time; 5 = all of the time |

**Covariables**

Chronic disease covariables for robustness checks included binary variables (yes, no (reference)) for hyper- and hypothyroidism; rheumatoid arthritis; asthma; CVD; cancer, osteoporosis; diabetes; Parkinson's; and stroke; and a binary variable for medications relevant to each chronic condition. Marital status covariable had 4 categories (married / living as married (reference group), never married, widowed, divorced/separated) based on 6 original response options questions (married, living as married, never married, widowed, divorced and separated). Despite being potential mediators between social support and CVRFs, multiple covariables of health behaviours were also included in robustness checks. Alcohol consumption frequency was dichotomized as weekly or more (every day, 4-5 times a week, 2-3 times a week, once a week) versus the reference group of less than weekly (2-3 times a month, about once a month, less than once a month or never) based on responses to the CLSA question “about how often during the past 12 months did you drink alcohol?” Sleep duration (hours) was based on the self-reported average number of hours of actual sleep CLSA participants had per night in the past month and sleep quality was defined as very satisfied or satisfied (reference) versus neutral, dissatisfied or very dissatisfied in response to the CLSA question “how satisfied or dissatisfied are you with your current sleep pattern?”. The amount of daily physical activity was measured as a Physical Activity Scale for the Elderly (PASE) score that was calculated from reported frequencies and durations of participation in 12 different activities over the previous week and then converted into hours-per-day values. Total times per day of combined fruits and vegetables was assessed using dietary data collected by CLSA’s Short Diet Questionnaire (a 36-iem validated food frequency questionnaire) which first asked whether they consumed fruits and four vegetables daily, weekly, monthly or yearly followed by the question ‘how many times’ for each frequency response (e.g. twice a day, three times a week, once a month); CLSA converted all SDQ questions into times per day. Three psychological factors were measured by the Center for Epidemiological Studies Depression scale (CESD) (score range 0-30); life close to ideal question from the Life Satisfaction Scale (agree (reference), neither, disagree) and depression medication (yes/no). Biological sex risk factors related to women’s reproductive status were the number of biological children (numeric), menopause status (yes (reference), no) and hormone replacement therapy use (ever (reference), never).

**Statistical analysis**

In order to avoid the multicollinearity issues between the correlated independent variables of social support, we used a four-step process of data analysis. Step 2 involved the selection of a subset of PCs based on the R squared and root mean squared error of prediction criteria; the PC retained were based on their contribution to variance explained and their ability to minimize RMSEP and thereby ensure robust model performance without overfitting. Below are S2 and S3 Tables that list the total number of PCs retained for use in each PCR model of continuous and binary outcomes, by gender. As a concrete example, S4 Table illustrates the PCA scores used for the PCR linear regression model of continuous BMI as an outcome among men in the CLSA.

**S2 Table. Number of PCs used in PCR models for continuous outcomes stratified by gender**

| **Outcome** | **Gender** | **# of PCs used in PCR** |
| --- | --- | --- |
| BMI | Men | 6 |
| WC | Men | 7 |
| SBP | Men | 6 |
| DBP | Men | 7 |
| BMI | Women | 8 |
| WC | Women | 8 |
| SBP | Women | 8 |
| DBP | Women | 9 |

**S3 Table. Number of PCs used in PCR models for binary outcomes stratified by gender**

| **Outcome** | **Gender** | **# of PCs used in PCR** |
| --- | --- | --- |
| Hypertension | Men | 8 |
| General obesity | Men | 8 |
| Central obesity | Men | 8 |
| Hypertension | Women | 7 |
| General obesity | Women | 8 |
| Central obesity | Women | 8 |

**S4 Table. PCA scores for the PCR model of the BMI outcome among men**

| **Type of social support** | **Components** | | | | | | | | | | |
| --- | --- | --- | --- | --- | --- | --- | --- | --- | --- | --- | --- |
|  | **PC1** | **PC2** | **PC3** | **PC4** | **PC5** | **PC6** | **PC7** | **PC8** | **PC9** | **PC10** |  |
| **Tangible** | -0.308 | -0.5 | 0.064 | 0.018 | 0.558 | -0.437 | 0.376 | -0.005 | -0.02 | 0.082 |  |
| **Emotional** | -0.336 | 0.248 | 0.298 | 0.592 | 0.218 | 0.191 | -0.169 | 0.097 | 0.412 | 0.305 |  |
| **Informational** | -0.335 | 0.258 | 0.39 | -0.491 | 0.29 | 0.236 | -0.194 | -0.079 | -0.461 | 0.177 |  |
| **Belonging** | -0.328 | 0.222 | -0.526 | -0.029 | 0.391 | 0.096 | -0.219 | -0.021 | 0.116 | -0.584 |  |
| **Tangible x Emotional** | -0.299 | -0.375 | 0.119 | 0.303 | -0.293 | -0.038 | -0.364 | -0.594 | -0.237 | -0.189 |  |
| **Tangible x Informational** | -0.297 | -0.366 | 0.183 | -0.314 | -0.275 | -0.084 | -0.381 | 0.558 | 0.296 | -0.138 |  |
| **Tangible x Belonging** | -0.293 | -0.348 | -0.359 | -0.032 | -0.143 | 0.69 | 0.318 | 0.045 | -0.033 | 0.249 |  |
| **Emotional x Informational** | -0.325 | 0.258 | 0.369 | 0.018 | -0.314 | 0.016 | 0.603 | 0.001 | 0.047 | -0.476 |  |
| **Emotional x Belonging** | -0.319 | 0.229 | -0.317 | 0.287 | -0.25 | -0.341 | -0.008 | 0.398 | -0.534 | 0.198 |  |
| **Informational x Belonging** | -0.317 | 0.241 | -0.255 | -0.365 | -0.245 | -0.325 | 0.042 | -0.399 | 0.414 | 0.381 |  |

**Supplementary Results**

**S5 Table. Adjusted means of anthropometric measures by functional social ties and their interactions in older women and men in CLSA (2011-15)**

|  | **Body mass index (kg/m^2^)** | | **Waist Circumference (cm)** | |
| --- | --- | --- | --- | --- |
|  | **Women** | **Men** | **Women** | **Men** |
| **Informational support** | | | | |
| High (20) | 27.42 (27.38, 27.45) | 28.2 (28.17, 28.23) | 87.21 (87.1, 87.32) | 99.75 (99.65, 99.85) |
| Low (4-19) | 27.93 (27.91, 27.95) | 28.29 (28.27, 28.31) | 88.64 (88.57, 88.71) | 100.34 (100.28, 100.4) |
| **Tangible support** | | | | |
| High (20) | 27.51 (27.47, 27.54) | 28.24 (28.21, 28.27) | 87.51 (87.39, 87.62) | 100.05 (99.96, 100.13) |
| Low (4-19) | 27.89 (27.87, 27.91) | 28.28 (28.26, 28.3) | 88.51 (88.44, 88.58) | 100.27 (100.2, 100.33) |
| **Emotional support** |  |  |  |  |
| High (35) | 27.44 (27.4, 27.47) | 28.26 (28.23, 28.3) | 87.1 (86.98, 87.22) | 100.07 (99.96, 100.17) |
| Low (7-34) | 27.9 (27.88, 27.93) | 28.27 (28.25, 28.29) | 88.61 (88.55, 88.68) | 100.23 (100.17, 100.29) |
| **Belonging support** | | | | |
| High (20) | 27.47 (27.44, 27.51) | 28.36 (28.33, 28.39) | 87.26 (87.15, 87.37) | 100.18 (100.09, 100.28) |
| Low (4-19) | 27.91 (27.89, 27.93) | 28.23 (28.21, 28.25) | 88.61 (88.54, 88.67) | 100.19 (100.13, 100.25) |
| **Informational and Tangible support** | | | | |
| High – High | 27.34 (27.30, 27.38) | 28.17 (28.14, 28.21) | 87.06 (86.92, 87.2) | 99.73 (99.61, 99.85) |
| High – Low | 27.55 (27.49, 27.6) | 28.27 (28.21, 28.33) | 87.46 (87.28, 87.64) | 99.82 (99.63, 100.01) |
| Low – High | 27.81 (27.75, 27.87) | 28.32 (28.28, 28.36) | 88.33 (88.14, 88.52) | 100.44 (100.31, 100.57) |
| Low – Low | 27.95 (27.93, 27.97) | 28.29 (28.26, 28.31) | 88.69 (88.62, 88.76) | 100.32 (100.25, 100.38) |
| **Informational and Emotional support** | | | | |
| High – High | 27.36 (27.32, 27.4) | 28.21 (28.17, 28.24) | 86.88 (86.75, 87.01) | 99.77 (99.65, 99.89) |
| High – Low | 27.56 (27.5, 27.62) | 28.18 (28.12, 28.24) | 88.00 (87.80, 88.19) | 99.69 (99.49, 99.88) |
| Low – High | 27.74 (27.66, 27.82) | 28.45 (28.39, 28.52) | 87.97 (87.70, 88.23) | 101.00 (100.79, 101.22) |
| Low – Low | 27.95 (27.93, 27.97) | 28.28 (28.26, 28.3) | 88.69 (88.62, 88.76) | 100.28 (100.22, 100.34) |
| **Informational and Belonging support** | | | | |
| High – High | 27.37 (27.33, 27.41) | 28.23 (28.2, 28.27) | 86.99 (86.86, 87.12) | 99.81 (99.69, 99.92) |
| High – Low | 27.55 (27.49, 27.61) | 28.08 (28.02, 28.15) | 87.77 (87.56, 87.98) | 99.55 (99.35, 99.76) |
| Low – High | 27.77 (27.7, 27.83) | 28.64 (28.59, 28.69) | 88.01 (87.8, 88.23) | 100.99 (100.83, 101.16) |
| Low – Low | 27.95 (27.93, 27.97) | 28.24 (28.22, 28.26) | 88.71 (88.64, 88.78) | 100.25 (100.19, 100.31) |
| **Tangible and Emotional support** | | | | |
| High – High | 27.33 (27.29, 27.38) | 28.21 (28.17, 28.24) | 86.96 (86.81, 87.11) | 99.91 (99.79, 100.04) |
| High – Low | 27.76 (27.7, 27.81) | 28.28 (28.24, 28.32) | 88.31 (88.13, 88.48) | 100.21 (100.08, 100.34) |
| Low – High | 27.62 (27.56, 27.68) | 28.44 (28.37, 28.5) | 87.36 (87.16, 87.56) | 100.53 (100.33, 100.74) |
| Low – Low | 27.93 (27.91, 27.95) | 28.27 (28.25, 28.29) | 88.67 (88.6, 88.74) | 100.24 (100.17, 100.3) |
| **Tangible and Belonging support** | | | | |
| High – High | 27.34 (27.3, 27.38) | 28.27 (28.24, 28.3) | 86.97 (86.83, 87.11) | 99.97 (99.86, 100.08) |
| High – Low | 27.82 (27.76, 27.88) | 28.19 (28.15, 28.23) | 88.51 (88.32, 88.7) | 100.17 (100.03, 100.31) |
| Low – High | 27.71 (27.65, 27.77) | 28.61 (28.55, 28.66) | 87.77 (87.59, 87.95) | 100.75 (100.58, 100.93) |
| Low – Low | 27.92 (27.9, 27.94) | 28.24 (28.22, 28.26) | 88.62 (88.55, 88.69) | 100.02 (100.13, 100.26) |
| **Belonging and Emotional support** | | | | |
| High – High | 27.41 (27.37, 27.45) | 28.27 (28.24, 28.31) | 87.02 (86.88, 87.15) | 99.99 (99.87, 100.11) |
| High – Low | 27.62 (27.56, 27.68) | 28.56 (28.5, 28.61) | 87.83 (87.63, 88.02) | 100.61 (100.45, 100.77) |
| Low – High | 27.54 (27.46, 27.61) | 28.24 (28.17, 28.3) | 87.39 (87.15, 87.64) | 100.36 (100.13, 100.59) |
| Low – Low | 27.94 (27.92, 27.96) | 28.23 (28.21, 28.25) | 88.71 (88.64, 88.78) | 100.18 (100.12, 100.24) |

**S6 Table. Joint associations between functional social ties and odds of general and central obesity in older women and men in CLSA (2011-15)**

|  | **General obesity model OR (CI95)** | | | **Central obesity model OR (CI95)** | |
| --- | --- | --- | --- | --- | --- |
| **Social support** | **Women** | **Men** | **Women** | | **Men** |
| **Informational** |  |  |  | |  |
| High (20) | Ref | Ref | Ref | | Ref |
| Low (4-19) | 1.07 (0.95, 1.2) | **1.21 (1.06, 1.38)^‡^** | 1.04 (0.91, 1.18) | | **1.16 (1.01, 1.34)^†^** |
| **Tangible** |  |  |  | |  |
| High (20) | Ref | Ref | Ref | | Ref |
| Low (4-19) | 0.97 (0.82, 1.15) | 1.09 (0.9, 1.32) | 1.16 (0.96, 1.39) | | 1.22 (1, 1.49) |
| **Emotional** |  |  |  | |  |
| High (35) | Ref | Ref | Ref | | Ref |
| Low (7-34) | 1.04 (0.89, 1.22) | 1.05 (0.93, 1.19) | 1.02 (0.86, 1.22) | | 1.01 (0.89, 1.16) |
| **Belonging** |  |  |  | |  |
| High (20) | Ref | Ref | Ref | | Ref |
| Low (4-19) | 1.09 (0.97, 1.22) | 1.08 (0.96, 1.22) | 1.03 (0.91, 1.17) | | 1.07 (0.94, 1.21) |
| **Informational+Tangible** |  |  |  | |  |
| High – High | Ref | Ref | Ref | | Ref |
| High – Low | Ref | Ref | Ref | | Ref |
| Low – High | Ref | Ref | Ref | | Ref |
| Low – Low | 1.12 (0.96, 1.32) | 0.92 (0.74, 1.14) | 1.04 (0.87, 1.24) | | 0.94 (0.75, 1.17) |
| **Informational+Emotional** |  |  |  | |  |
| High – High | Ref | Ref | Ref | | Ref |
| High – Low | Ref | Ref | Ref | | Ref |
| Low – High | Ref | Ref | Ref | | Ref |
| Low – Low | 0.91 (0.74, 1.12) | 0.9 (0.75, 1.08) | 0.93 (0.74, 1.17) | | 0.9 (0.74, 1.1) |
| **Informational+Belonging** |  |  |  | |  |
| High – High | Ref | Ref | Ref | | Ref |
| High – Low | Ref | Ref | Ref | | Ref |
| Low – High | Ref | Ref | Ref | | Ref |
| Low – Low | 0.92 (0.73, 1.16) | 1.06 (0.9, 1.25) | 1.06 (0.82, 1.37) | | 1.06 (0.89, 1.26) |
| **Tangible+Emotional** |  |  |  | |  |
| High – High | Ref | Ref | Ref | | Ref |
| High – Low | Ref | Ref | Ref | | Ref |
| Low – High | Ref | Ref | Ref | | Ref |
| Low – Low | 1 (0.9, 1.1) | 1.17 (0.94, 1.46) | 0.98 (0.88, 1.1) | | 1.09 (0.87, 1.38) |
| **Tangible+Belonging** |  |  |  | |  |
| High – High | Ref | Ref | Ref | | Ref |
| High – Low | Ref | Ref | Ref | | Ref |
| Low – High | Ref | Ref | Ref | | Ref |
| Low – Low | 0.92 (0.78, 1.09) | 0.89 (0.73, 1.09) | 0.85 (0.71, 1.02) | | 0.84 (0.68, 1.04) |
| **Belonging+Emotional** |  |  |  | |  |
| High – High | Ref | Ref | Ref | | Ref |
| High – Low | Ref | Ref | Ref | | Ref |
| Low – High | Ref | Ref | Ref | | Ref |
| Low – Low | 1.16 (0.94, 1.43) | 0.83 (0.71, 0.98) | 1.16 (0.92, 1.45) | | 0.87 (0.73, 1.03) |

**S7 Table. Adjusted means of blood pressure by functional social ties and their interactions in older women and men in CLSA (2011-15)**

|  | **Systolic blood pressure (SBP, mm Hg)** | | **Diastolic blood pressure (DBP, mm Hg)** | |
| --- | --- | --- | --- | --- |
|  | **Women** | **Men** | **Women** | **Men** |
| **Informational support** | | | | |
| High (20) | 118.5 (118.3, 118.7) | 122.0 (121.9, 122.1) | 72.0 (71.9, 72.0) | 76.2 (76.1, 76.3) |
| Low (4-19) | 120.0 (119.8, 120.1) | 122.6 (122.5, 122.7) | 71.6 (71.6, 71.7) | 76.1 (76.0, 76.1) |
| **Tangible support** | | | | |
| High (20) | 119.1 (118.9, 119.3) | 122.0 (121.9, 122.1) | 71.7 (71.6, 71.7) | 75.8 (75.7, 75.9) |
| Low (4-19) | 119.7 (119.6, 119.9) | 122.7 (122.6, 122.77) | 71.8 (71.7, 71.8) | 76.3 (76.2, 76.3) |
| **Emotional support** |  |  |  |  |
| High (35) | 118.6 (118.4, 118.8) | 121.9 (121.8, 122.0) | 72.0 (71.9, 72.0) | 76.1 (76.0, 76.2) |
| Low (7-34) | 119.9 (119.8, 120.0) | 122.6 (122.6, 122.7) | 71.6 (71.6, 71.7) | 76.1 (76.0, 76.2) |
| **Belonging support** | | | | |
| High (20) | 118.9 (118.7, 119.1) | 122.3 (122.2, 122.4) | 71.9 (71.9, 72.0) | 76.1 (76.0, 76.2) |
| Low (4-19) | 119.8 (119.7, 119.9) | 122.5 (122.4, 122.6) | 71.7 (71.6, 71.7) | 76.1 (76.0, 76.2) |
| **Informational and Tangible support** | | | | |
| High – High | 118.6 (118.3, 118.8) | 121.9 (121.7, 122.0) | 71.8 (71.8, 71.9) | 76.0 (75.9, 76.2) |
| High – Low | 118.5 (118.2, 118.9) | 122.3 (122.1, 122.6) | 72.2 (72.1, 72.3) | 76.7 (76.5, 76.9) |
| Low – High | 120.0 (119.6, 120.3) | 122.1 (122.0, 122.3) | 71.3 (71.2, 71.4) | 75.5 (75.4, 75.6) |
| Low – Low | 120.0 (119.8, 120.1) | 122.7 (122.7, 122.8) | 71.7 (71.6, 71.7) | 76.2 (76.1, 76.3) |
| **Informational and Emotional support** | | | | |
| High – High | 118.2 (118.0, 118.4) | 121.9 (121.7, 122.0) | 71.9 (71.9, 72.0) | 76.1 (76.0, 76.2) |
| High – Low | 119.3 (119.0, 119.7) | 122.3 (122.1, 122.6) | 72.1 (72.0, 72.2) | 76.5 (76.3, 76.8) |
| Low – High | 120.1 (119.6, 120.6) | 122.0 (121.7, 122.2) | 72.2 (72.1, 72.4) | 76.0 (75.7, 76.2) |
| Low – Low | 119.9 (119.8, 120.1) | 122.7 (122.6, 122.7) | 71.6 (71.6, 71.6) | 76.1 (76.0, 76.1) |
| **Informational and Belonging support** | | | | |
| High – High | 118.5 (118.3, 118.8) | 122.1 (122.0, 122.2) | 71.9 (71.8, 72.0) | 76.1 (76.0, 76.2) |
| High – Low | 118.6 (118.2, 118.9) | 121.7 (121.4, 121.9) | 72.2 (72.0, 72.3) | 76.5 (76.3, 76.8) |
| Low – High | 119.9 (119.5, 120.4) | 122.8 (122.6, 123.1) | 72.0 (71.9, 72.1) | 76.1 (76.0, 76.3) |
| Low – Low | 120.0 (119.8, 120.1) | 122.6 (122.5, 122.7) | 71.6 (71.6, 71.6) | 76.1 (76.0, 76.1) |
| **Tangible and Emotional support** | | | | |
| High – High | 118.5 (118.2, 118.8) | 121.8 (121.7, 122.0) | 71.8 (71.8, 71.9) | 75.9 (75.8, 76.0) |
| High – Low | 119.9 (119.6, 120.2) | 122.2 (122.0, 122.4) | 71.4 (71.3, 71.5) | 75.6 (75.5, 75.8) |
| Low – High | 118.8 (118.4, 119.1) | 122.2 (121.9, 122.4) | 72.3 (72.2, 72.3) | 76.5 (76.3, 76.7) |
| Low – Low | 119.9 (119.7, 120.0) | 122.7 (122.7, 122.8) | 71.7 (71.6, 71.7) | 76.2 (76.2, 76.3) |
| **Tangible and Belonging support** | | | | |
| High – High | 118.5 (118.2, 118.7) | 122.0 (121.9, 122.1) | 71.8 (71.8, 71.9) | 76.0 (75.8, 76.1) |
| High – Low | 120.1 (119.8, 120.5) | 121.9 (121.8, 122.1) | 71.4 (71.3, 71.5) | 75.5 (75.4, 75.7) |
| Low – High | 119.7 (119.3, 120.0) | 123.2 (123.0, 123.4) | 72.1 (72.0, 72.2) | 76.6 (76.4, 76.8) |
| Low – Low | 119.8 (119.6, 119.9) | 122.6 (122.5, 122.7) | 71.7 (71.7, 71.7) | 76.2 (76.1, 76.3) |
| **Belonging and Emotional support** | | | | |
| High – High | 118.5 (118.3, 118.8) | 122.0 (121.9, 122.2) | 71.9 (71.9, 72.0) | 76.0 (75.9, 76.2) |
| High – Low | 119.8 (119.4, 120.1) | 123.0 (122.8, 123.2) | 71.9 (71.8, 72.0) | 76.3 (76.2, 76.5) |
| Low – High | 118.8 (118.3, 119.2) | 121.4 (121.1, 121.7) | 72.2 (72.1, 72.3) | 76.2 (76.0, 76.4) |
| Low – Low | 119.9 (119.8, 120.0) | 122.6 (122.5, 122.7) | 71.6 (71.6, 71.7) | 76.1 (76.0, 76.2) |

**S8 Table. Joint associations between functional social ties and odds of hypertension in older women and men in CLSA (2011-15)**

|  | **Odds ratios (CI95)** | |
| --- | --- | --- |
|  | **Women** | **Men** |
| **Informational support** | | |
| High (20) | Ref | Ref |
| Low (4-19) | 1.03 (0.91, 1.15) | 1.09 (0.95, 1.25) |
| **Tangible support** | | |
| High (20) | Ref | Ref |
| Low (4-19) | 1.05 (0.88, 1.26) | 0.97 (0.8, 1.18) |
| **Emotional support** | | |
| High (35) | Ref | Ref |
| Low (7-34) | 1.03 (0.91, 1.17) | 1.06 (0.94, 1.21) |
| **Belonging support** | | |
| High (20) | Ref | Ref |
| Low (4-19) | 1 (0.89, 1.12) | 1.06 (0.94, 1.19) |
| **Informational and Tangible support** | | |
| High – High | Ref | Ref |
| High – Low | Ref | Ref |
| Low – High | Ref | Ref |
| Low – Low | 1.05 (0.90, 1.23) | 0.97 (0.78, 1.21) |
| **Informational and Emotional support** | | |
| High – High | Ref | Ref |
| High – Low | Ref | Ref |
| Low – High | Ref | Ref |
| Low – Low | 0.94 (0.77, 1.16) | 0.91 (0.76, 1.10) |
| **Informational and Belonging support** | | |
| High – High | Ref | Ref |
| High – Low | Ref | Ref |
| Low – High | Ref | Ref |
| Low – Low | 1.06 (0.94, 1.20) | 1.09 (0.93, 1.29) |
| **Tangible and Emotional support** | | |
| High – High | Ref | Ref |
| High – Low | Ref | Ref |
| Low – High | Ref | Ref |
| Low – Low | 1.01 (0.92, 1.11) | 1.17 (0.94, 1.47) |
| **Tangible and Belonging support** | | |
| High – High | Ref | Ref |
| High – Low | Ref | Ref |
| Low – High | Ref | Ref |
| Low – Low | 0.89 (0.75, 1.07) | 0.84 (0.68, 1.03) |
| **Belonging and Emotional support** | | |
| High – High | Ref | Ref |
| High – Low | Ref | Ref |
| Low – High | Ref | Ref |
| Low – Low | 1.09 (0.94, 1.26) | 0.97 (0.82, 1.15) |

**S9 Table. Sensitivity analysis of joint associations between functional social ties and adjusted mean BMI in older women in CLSA (2011-15).**

|  | **Model A: final + behaviours** | | **Model B: final + psychological** | | | **Model C: final + marital status** | | | **Model D: final + chronic disease** | | | **Model E: final + blood pressure** | | | **Model F: final + reproductive** | | |
| --- | --- | --- | --- | --- | --- | --- | --- | --- | --- | --- | --- | --- | --- | --- | --- | --- | --- |
| **Informational support** | | | |  | | |  | | |  | | |  | | |  |  |
| High (20) | 27.41  (27.36, 27.47) | | 27.41  (27.36, 27.47) | | | 27.42  (27.38, 27.45) | | | 27.42  (27.38, 27.45) | | | 27.42  (27.36, 27.49) | | | 27.35  (27.31, 27.39) | | |
| Low (4-19) | 27.89  (27.86, 27.93) | | 27.89  (27.86, 27.93) | | | 27.93  (27.91, 27.96) | | | 27.93  (27.91, 27.96) | | | 27.93  (27.89, 27.97) | | | 27.87  (27.85, 27.9) | | |
| **Tangible support** | |  | | |  | | |  | | |  | | |  | | |  |
| High (20) | 27.49  (27.43, 27.54) | | 27.51  (27.47, 27.55) | | | 27.5  (27.47, 27.54) | | | 27.5  (27.47, 27.54) | | | 27.51  (27.44, 27.57) | | | 27.46  (27.42, 27.5) | | |
| Low (4-19) | 27.86 (27.83, 27.89) | | 27.9  (27.87, 27.92) | | | 27.89  (27.87, 27.92) | | | 27.89  (27.87, 27.92) | | | 27.89  (27.85, 27.93) | | | 27.83  (27.8, 27.85) | | |
| **Emotional support** | |  | | |  | | |  | | |  | | |  | | |  |
| High (35) | 27.42  (27.36, 27.47) | | 27.44  (27.4, 27.48) | | | 27.44  (27.4, 27.48) | | | 27.44  (27.4, 27.48) | | | 27.44  (27.37, 27.51) | | | 27.41  (27.37, 27.44) | | |
| Low (7-34) | 27.87  (27.84, 27.91) | | 27.91  (27.88, 27.93) | | | 27.9  (27.88, 27.93) | | | 27.9  (27.88, 27.93) | | | 27.9  (27.87, 27.94) | | | 27.83  (27.81, 27.86) | | |
| **Belonging support** | |  | | |  | | |  | | |  | | |  | | |  |
| High (20) | 27.46  (27.41, 27.52) | | 27.48  (27.44, 27.52) | | | 27.47  (27.44, 27.51) | | | 27.47  (27.44, 27.51) | | | 27.47  (27.4, 27.53) | | | 27.43  (27.39, 27.47) | | |
| Low (4-19) | 27.87  (27.84, 27.91) | | 27.91  (27.88, 27.94) | | | 27.91  (27.88, 27.93) | | | 27.91  (27.88, 27.93) | | | 27.91  (27.87, 27.95) | | | 27.84  (27.82, 27.86) | | |
| **Informational-Tangible support** | | | |  | | |  | | |  | | |  | | |  |  |
| High – High | 27.33  (27.25, 27.4) | | 27.35  (27.3, 27.4) | | | 27.34  (27.29, 27.38) | | | 27.34  (27.29, 27.38) | | | 27.35  (27.27, 27.43) | | | 27.29  (27.25, 27.34) | | |
| High – Low | 27.55  (27.46, 27.63) | | 27.54  (27.48, 27.61) | | | 27.55  (27.49, 27.61) | | | 27.55  (27.49, 27.61) | | | 27.54  (27.44, 27.64) | | | 27.44  (27.39, 27.5) | | |
| Low – High | 27.77  (27.68, 27.87) | | 27.79  (27.73, 27.86) | | | 27.81  (27.74, 27.87) | | | 27.81  (27.74, 27.87) | | | 27.8  (27.69, 27.91) | | | 27.76  (27.7, 27.82) | | |
| Low – Low | 27.91  (27.88, 27.95) | | 27.96  (27.93, 27.98) | | | 27.95  (27.93, 27.98) | | | 27.95  (27.93, 27.98) | | | 27.95  (27.91, 27.99) | | | 27.89  (27.87, 27.92) | | |
| **Informational-Emotional support** | |  | | |  | | |  | | |  | | |  | | |  |
| High – High | 27.35  (27.28, 27.42) | | 27.37  (27.32, 27.41) | | | 27.36  (27.31, 27.4) | | | 27.36  (27.31, 27.4) | | | 27.37  (27.29, 27.44) | | | 27.34  (27.3, 27.38) | | |
| High – Low | 27.55  (27.45, 27.65) | | 27.56  (27.49, 27.64) | | | 27.56  (27.49, 27.63) | | | 27.56  (27.49, 27.63) | | | 27.55  (27.43, 27.67) | | | 27.37  (27.31, 27.44) | | |
| Low – High | 27.66  (27.53, 27.79) | | 27.73  (27.63, 27.82) | | | 27.75  (27.66, 27.84) | | | 27.75  (27.66, 27.84) | | | 27.71  (27.56, 27.87) | | | 27.66  (27.58, 27.75) | | |
| Low – Low | 27.91  (27.88, 27.95) | | 27.95  (27.92, 27.98) | | | 27.95  (27.92, 27.97) | | | 27.95  (27.92, 27.97) | | | 27.95  (27.91, 27.99) | | | 27.89  (27.87, 27.91) | | |
| **Informational-Belonging support** | |  | | |  | | |  | | |  | | |  | | |  |
| High – High | 27.36  (27.29, 27.42) | | 27.37  (27.33, 27.42) | | | 27.37  (27.32, 27.41) | | | 27.37  (27.32, 27.41) | | | 27.37  (27.29, 27.44) | | | 27.33  (27.29, 27.37) | | |
| High – Low | 27.55  (27.45, 27.65) | | 27.55  (27.48, 27.63) | | | 27.55  (27.48, 27.62) | | | 27.55  (27.48, 27.62) | | | 27.56  (27.45, 27.68) | | | 27.4  (27.33, 27.46) | | |
| Low – High | 27.75  (27.64, 27.86) | | 27.76  (27.69, 27.84) | | | 27.77  (27.7, 27.84) | | | 27.77  (27.7, 27.84) | | | 27.76  (27.63, 27.88) | | | 27.71  (27.64, 27.77) | | |
| Low – Low | 27.91  (27.87, 27.95) | | 27.95  (27.93, 27.98) | | | 27.95  (27.93, 27.97) | | | 27.95  (27.93, 27.97) | | | 27.95  (27.91, 27.99) | | | 27.89  (27.87, 27.92) | | |
| **Tangible-Emotional support** | |  | | |  | | |  | | |  | | |  | | |  |
| High – High | 27.31  (27.23, 27.38) | | 27.33  (27.28, 27.38) | | | 27.33  (27.29, 27.38) | | | 27.33  (27.29, 27.38) | | | 27.34  (27.25, 27.42) | | | 27.31  (27.26, 27.36) | | |
| High – Low | 27.75  (27.66, 27.83) | | 27.76  (27.7, 27.83) | | | 27.75  (27.69, 27.81) | | | 27.75  (27.69, 27.81) | | | 27.75  (27.65, 27.85) | | | 27.68  (27.62, 27.73) | | |
| Low – High | 27.61  (27.51, 27.7) | | 27.64  (27.56, 27.71) | | | 27.62  (27.56, 27.69) | | | 27.62  (27.56, 27.69) | | | 27.62  (27.5, 27.73) | | | 27.58  (27.51, 27.64) | | |
| Low – Low | 27.89  (27.86, 27.93) | | 27.93  (27.9, 27.96) | | | 27.93  (27.91, 27.95) | | | 27.93  (27.91, 27.95) | | | 27.93  (27.89, 27.97) | | | 27.86  (27.84, 27.88) | | |
| **Tangible-Belonging support** | |  | | |  | | |  | | |  | | |  | | |  |
| High – High | 27.32  (27.25, 27.39) | | 27.34  (27.29, 27.38) | | | 27.34  (27.3, 27.39) | | | 27.34  (27.3, 27.39) | | | 27.33  (27.25, 27.41) | | | 27.32  (27.28, 27.37) | | |
| High – Low | 27.8  (27.71, 27.89) | | 27.82  (27.75, 27.89) | | | 27.8  (27.74, 27.87) | | | 27.8  (27.74, 27.87) | | | 27.84  (27.73, 27.95) | | | 27.71  (27.65, 27.77) | | |
| Low – High | 27.72  (27.62, 27.81) | | 27.72  (27.66, 27.78) | | | 27.71  (27.65, 27.77) | | | 27.71  (27.65, 27.77) | | | 27.72  (27.61, 27.83) | | | 27.62  (27.56, 27.68) | | |
| Low – Low | 27.88  (27.85, 27.92) | | 27.92  (27.9, 27.95) | | | 27.92  (27.9, 27.95) | | | 27.92  (27.9, 27.95) | | | 27.92  (27.88, 27.96) | | | 27.86  (27.83, 27.88) | | |
| **Belonging-Emotional support** | |  | | |  | | |  | | |  | | |  | | |  |
| High – High | 27.39  (27.32, 27.46) | | 27.41  (27.36, 27.45) | | | 27.41  (27.37, 27.45) | | | 27.41  (27.37, 27.45) | | | 27.4  (27.32, 27.48) | | | 27.38  (27.34, 27.43) | | |
| High – Low | 27.63  (27.53, 27.73) | | 27.64  (27.57, 27.71) | | | 27.62  (27.56, 27.69) | | | 27.62  (27.56, 27.69) | | | 27.63  (27.51, 27.75) | | | 27.54  (27.48, 27.61) | | |
| Low – High | 27.5  (27.38, 27.62) | | 27.55  (27.46, 27.65) | | | 27.53  (27.44, 27.61) | | | 27.53  (27.44, 27.61) | | | 27.57  (27.43, 27.71) | | | 27.49  (27.41, 27.57) | | |
| Low – Low | 27.9  (27.87, 27.94) | | 27.94  (27.91, 27.97) | | | 27.94  (27.91, 27.96) | | | 27.94  (27.91, 27.96) | | | 27.94  (27.9, 27.98) | | | 27.87  (27.85, 27.89) | | |

Model A is the final main model (age, age2, education, smoking, province) plus health behaviours (alcohol, diet, physical activity and sleep)

Model B is the final main model (age, age2, education, smoking, province) plus psychological factors

Model C is the final main model (age, age2, education, smoking, province) plus marital status

Model D is the final main model (age, age2, education, smoking, province) plus chronic conditions

Model E is the final main model (age, age2, education, smoking, province) plus blood pressure (systolic blood pressure and diastolic blood pressure)

Model F is the final main model (age, age2, education, smoking, province) plus women’s reproductive status (parity, menopause status, hormone replacement therapy)

**S10 Table. Sensitivity analysis of joint associations between functional social ties and adjusted mean BMI in older men in CLSA (2011-15).**

|  | **Model A: final + behaviours** | **Model B: final + psychological** | **Model C: final + marital status** | **Model D: final + chronic disease** | **Model F: final + blood pressure** |
| --- | --- | --- | --- | --- | --- |
| **Informational support** | |  |  |  |  |
| High (20) | 28.18  (28.14, 28.22) | 28.2  (28.17, 28.24) | 28.2  (28.17, 28.23) | 28.2  (28.17, 28.23) | 28.2  (28.15, 28.25) |
| Low (4-19) | 28.29  (28.27, 28.31) | 28.29  (28.27, 28.31) | 28.29  (28.28, 28.31) | 28.29  (28.28, 28.31) | 28.29  (28.26, 28.32) |
| **Tangible support** | |  |  |  |  |
| High (20) | 28.21  (28.18, 28.25) | 28.24  (28.21, 28.27) | 28.24  (28.21, 28.27) | 28.24  (28.21, 28.27) | 28.24  (28.2, 28.28) |
| Low (4-19) | 28.29 (  28.27, 28.32) | 28.28  (28.26, 28.3) | 28.29  (28.27, 28.31) | 28.29  (28.27, 28.31) | 28.28  (28.25, 28.32) |
| **Emotional support** | |  |  |  |  |
| High (35) | 28.25  (28.21, 28.29) | 28.27  (28.23, 28.3) | 28.26  (28.23, 28.3) | 28.26  (28.23, 28.3) | 28.26  (28.22, 28.31) |
| Low (7-34) | 28.27  (28.24, 28.29) | 28.27  (28.25, 28.29) | 28.27  (28.25, 28.29) | 28.27  (28.25, 28.29) | 28.27  (28.24, 28.3) |
| **Belonging support** | |  |  |  |  |
| High (20) | 28.35  (28.31, 28.39) | 28.36  (28.33, 28.39) | 28.36  (28.33, 28.39) | 28.36  (28.33, 28.39) | 28.36  (28.32, 28.41) |
| Low (4-19) | 28.23  (28.2, 28.25) | 28.23  (28.21, 28.25) | 28.23  (28.21, 28.25) | 28.23  (28.21, 28.25) | 28.23  (28.2, 28.26) |
| **Informational-Tangible support** | |  |  |  |  |
| High – High | 28.15  (28.1, 28.2) | 28.18  (28.14, 28.22) | 28.17  (28.14, 28.21) | 28.17  (28.14, 28.21) | 28.17  (28.12, 28.23) |
| High – Low | 28.29  (28.21, 28.36) | 28.27  (28.2, 28.33) | 28.27  (28.21, 28.33) | 28.27  (28.21, 28.33) | 28.27  (28.18, 28.36) |
| Low – High | 28.29  (28.24, 28.34) | 28.32  (28.28, 28.36) | 28.32  (28.28, 28.36) | 28.32  (28.28, 28.36) | 28.32  (28.26, 28.38) |
| Low – Low | 28.29  (28.27, 28.32) | 28.28  (28.26, 28.3) | 28.29  (28.27, 28.31) | 28.29  (28.27, 28.31) | 28.29  (28.25, 28.32) |
| **Informational-Emotional support** | |  |  |  |  |
| High – High | 28.19  (28.15, 28.24) | 28.21  (28.17, 28.25) | 28.21  (28.17, 28.24) | 28.21  (28.17, 28.24) | 28.21  (28.15, 28.27) |
| High – Low | 28.16  (28.08, 28.24) | 28.18  (28.12, 28.25) | 28.18  (28.11, 28.24) | 28.18  (28.11, 28.24) | 28.17  (28.08, 28.26) |
| Low – High | 28.43  (28.35, 28.52) | 28.45  (28.38, 28.52) | 28.45  (28.38, 28.51) | 28.45  (28.38, 28.51) | 28.44  (28.34, 28.53) |
| Low – Low | 28.28  (28.25, 28.3) | 28.28  (28.25, 28.3) | 28.28  (28.26, 28.3) | 28.28  (28.26, 28.3) | 28.28  (28.25, 28.31) |
| **Informational-Belonging support** | |  |  |  |  |
| High – High | 28.22  (28.18, 28.27) | 28.24  (28.2, 28.27) | 28.23  (28.2, 28.27) | 28.23  (28.2, 28.27) | 28.23  (28.18, 28.29) |
| High – Low | 28.05  (27.97, 28.13) | 28.09  (28.02, 28.15) | 28.09  (28.02, 28.15) | 28.09  (28.02, 28.15) | 28.08  (27.99, 28.18) |
| Low – High | 28.62  (28.55, 28.69) | 28.64  (28.58, 28.69) | 28.64  (28.59, 28.69) | 28.64  (28.59, 28.69) | 28.64  (28.56, 28.72) |
| Low – Low | 28.24  (28.22, 28.27) | 28.24  (28.22, 28.26) | 28.25  (28.23, 28.27) | 28.25  (28.23, 28.27) | 28.24  (28.21, 28.27) |
| **Tangible-Emotional support** | |  |  |  |  |
| High – High | 28.19  (28.14, 28.23) | 28.21  (28.17, 28.24) | 28.21  (28.17, 28.24) | 28.21  (28.17, 28.24) | 28.2  (28.14, 28.25) |
| High – Low | 28.24  (28.19, 28.29) | 28.29  (28.25, 28.33) | 28.28  (28.24, 28.32) | 28.28  (28.24, 28.32) | 28.29  (28.23, 28.35) |
| Low – High | 28.44  (28.36, 28.52) | 28.45  (28.38, 28.52) | 28.44  (28.37, 28.5) | 28.44  (28.37, 28.5) | 28.46  (28.37, 28.56) |
| Low – Low | 28.28  (28.25, 28.3) | 28.26  (28.24, 28.28) | 28.27  (28.25, 28.29) | 28.27  (28.25, 28.29) | 28.27  (28.23, 28.3) |
| **Tangible-Belonging support** | |  |  |  |  |
| High – High | 28.25  (28.21, 28.29) | 28.28  (28.24, 28.31) | 28.27  (28.24, 28.3) | 28.27  (28.24, 28.3) | 28.27  (28.22, 28.32) |
| High – Low | 28.15  (28.09, 28.2) | 28.19  (28.14, 28.24) | 28.19  (28.15, 28.23) | 28.19  (28.15, 28.23) | 28.19  (28.12, 28.25) |
| Low – High | 28.62  (28.54, 28.69) | 28.6  (28.54, 28.66) | 28.61  (28.55, 28.66) | 28.61  (28.55, 28.66) | 28.61  (28.52, 28.69) |
| Low – Low | 28.25  (28.22, 28.27) | 28.24  (28.21, 28.26) | 28.24  (28.22, 28.26) | 28.24  (28.22, 28.26) | 28.24  (28.21, 28.27) |
| **Belonging-Emotional support** | |  |  |  |  |
| High – High | 28.26  (28.22, 28.31) | 28.28  (28.24, 28.31) | 28.27  (28.24, 28.31) | 28.27  (28.24, 28.31) | 28.28  (28.22, 28.33) |
| High – Low | 28.54  (28.47, 28.6) | 28.55  (28.5, 28.61) | 28.56  (28.5, 28.61) | 28.56  (28.5, 28.61) | 28.55  (28.47, 28.63) |
| Low – High | 28.2  (28.11, 28.28) | 28.23  (28.16, 28.3) | 28.24  (28.17, 28.31) | 28.24  (28.17, 28.31) | 28.22  (28.12, 28.32) |
| Low – Low | 28.23  (28.21, 28.26) | 28.23  (28.21, 28.25) | 28.23  (28.21, 28.25) | 28.23  (28.21, 28.25) | 28.23  (28.2, 28.26) |

Model A is the final main model (age, age2, education, smoking, province) plus health behaviours (alcohol, diet, physical activity and sleep)

Model B is the final main model (age, age2, education, smoking, province) plus psychological factors

Model C is the final main model (age, age2, education, smoking, province) plus marital status

Model D is the final main model (age, age2, education, smoking, province) plus chronic conditions

Model E is the final main model (age, age2, education, smoking, province) plus blood pressure (systolic blood pressure and diastolic blood pressure).

**S11 Table. Sensitivity analysis of joint associations between functional social ties and adjusted mean WC in older women in CLSA (2011-15).**

|  | **Model A: final + behaviours** | | **Model B: final + psychological** | | **Model C: final + marital status** | | **Model D: final + chronic disease** | | **Model E: final + blood pressure** | | **Model F: final + reproductive** | |
| --- | --- | --- | --- | --- | --- | --- | --- | --- | --- | --- | --- | --- |
| **Informational support** |  | |  | |  | |  | |  | |  | |
| High (20) | 87.2 (87, 87.3) | | 87.2 (87, 87.3) | | 87.2 (87.1, 87.3) | | 87.2 (87.1, 87.3) | | 87.2 (87.1, 87.4) | | 87.1 (87, 87.2) | |
| Low (4-19) | 88.5 (88.4, 88.6) | | 88.5 (88.4, 88.6) | | 88.6 (88.6, 88.7) | | 88.6 (88.6, 88.7) | | 88.6 (88.5, 88.7) | | 88.6 (88.5, 88.6) | |
| **Tangible support** |  | |  | |  | |  | |  | |  | |
| High (20) | 87.4 (87.3, 87.6) | | 87.5 (87.4, 87.6) | | 87.5 (87.4, 87.6) | | 87.5 (87.4, 87.6) | | 87.5 (87.3, 87.7) | | 87.4 (87.3, 87.5) | |
| Low (4-19) | 88.4 (88.3, 88.5) | | 88.5 (88.4, 88.6) | | 88.5 (88.4, 88.6) | | 88.5 (88.4, 88.6) | | 88.5 (88.4, 88.6) | | 88.4 (88.4, 88.5) | |
| **Emotional support** |  | |  | |  | |  | |  | |  | |
| High (35) | 87.0 (86.9, 87.2) | | 87.1 (87, 87.2) | | 87.1 (87, 87.2) | | 87.1 (87, 87.2) | | 87.1 (87, 87.3) | | 87 (87, 87.1) | |
| Low (7-34) | 88.5 (88.4, 88.6) | | 88.6 (88.6, 88.7) | | 88.6 (88.6, 88.7) | | 88.6 (88.6, 88.7) | | 88.6 (88.5, 88.7) | | 88.5 (88.5, 88.6) | |
| **Belonging support** |  | |  | |  | |  | |  | |  | |
| High (20) | 87.2 (87.0, 87.3) | | 87.3 (87.1, 87.4) | | 87.3 (87.2, 87.4) | | 87.3 (87.2, 87.4) | | 87.3 (87.1, 87.4) | | 87.1 (87.0, 87.3) | |
| Low (4-19) | 88.5 (88.4, 88.6) | | 88.6 (88.5, 88.7) | | 88.6 (88.5, 88.7) | | 88.6 (88.54, 88.7) | | 88.6 (88.5, 88.7) | | 88.5 (88.5, 88.6) | |
| **Informational-Tangible support** | |  | |  | |  | |  | |  | |  |
| High – High | 87 (86.8, 87.1) | | 87.1 (86.9, 87.3) | | 87.1 (86.9, 87.2) | | 87.1 (86.9, 87.2) | | 87.1 (86.9, 87.3) | | 86.9 (86.8, 87.1) | |
| High – Low | 87.4 (87.2, 87.7) | | 87.5 (87.3, 87.7) | | 87.5 (87.3, 87.7) | | 87.5 (87.3, 87.7) | | 87.5 (87.2, 87.7) | | 87.3 (87.1, 87.5) | |
| Low – High | 88.2 (88, 88.5) | | 88.3 (88.1, 88.5) | | 88.3 (88.1, 88.5) | | 88.3 (88.1, 88.5) | | 88.3 (88, 88.6) | | 88.3 (88.1, 88.5) | |
| Low – Low | 88.6 (88.5, 88.7) | | 88.7 (88.6, 88.8) | | 88.7 (88.6, 88.8) | | 88.7 (88.6, 88.8) | | 88.7 (88.6, 88.8) | | 88.6 (88.6, 88.7) | |
| **Informational-Emotional support** | |  | |  | |  | |  | |  | |  |
| High – High | 86.8 (86.6, 87) | | 86.9 (86.8, 87.1) | | 86.9 (86.7, 87.0) | | 86.9 (86.7, 87.0) | | 86.9 (86.7, 87.1) | | 86.8 (86.6, 86.9) | |
| High – Low | 87.9 (87.7, 88.2) | | 88.0 (87.8, 88.2) | | 88 (87.8, 88.2) | | 88 (87.8, 88.2) | | 88 (87.7, 88.3) | | 87.8 (87.6, 88.0) | |
| Low – High | 87.8 (87.5, 88.2) | | 87.9 (87.6, 88.2) | | 88 (87.7, 88.3) | | 88 (87.7, 88.3) | | 87.9 (87.5, 88.3) | | 87.9 (87.6, 88.1) | |
| Low – Low | 88.6 (88.5, 88.7) | | 88.7 (88.6, 88.8) | | 88.7 (88.6, 88.8) | | 88.7 (88.6, 88.8) | | 88.7 (88.6, 88.8) | | 88.6 (88.6, 88.7) | |
| **Informational-Belonging support** | |  | |  | |  | |  | |  | |  |
| High – High | **86.9 (86.7, 87.1)** | | 87.0 (86.9, 87.2) | | 87 (86.9, 87.1) | | 87 (86.9, 87.1) | | 87 (86.8, 87.2) | | 86.9 (86.8, 87.0) | |
| High – Low | **87.8 (87.5, 88.1)** | | 87.8 (87.6, 88.0) | | 87.8 (87.5, 88) | | 87.8 (87.5, 88) | | 87.8 (87.5, 88.1) | | 87.6 (87.4, 87.8) | |
| Low – High | **87.9 (87.6, 88.2)** | | 88 (87.7, 88.2) | | 88.0 (87.8, 88.3) | | 88.0 (87.8, 88.3) | | 88 (87.7, 88.3) | | 87.8 (87.6, 88.0) | |
| Low – Low | **88.6 (88.5, 88.7)** | | 88.7 (88.6, 88.8) | | 88.7 (88.6, 88.8) | | 88.7 (88.6, 88.8) | | 88.7 (88.6, 88.8) | | 88.7 (88.6, 88.7) | |
| **Tangible-Emotional support** | |  | |  | |  | |  | |  | |  |
| High – High | 86.9 (86.7, 87.1) | | 87 (86.8, 87.1) | | 87 (86.8, 87.1) | | 87 (86.8, 87.1) | | 87 (86.7, 87.2) | | 86.9 (86.7, 87.0) | |
| High – Low | 88.3 (88.0, 88.5) | | 88.3 (88.1, 88.5) | | 88.3 (88.1, 88.5) | | 88.3 (88.1, 88.5) | | 88.3 (88.0, 88.6) | | 88.2 (88.0, 88.4) | |
| Low – High | 87.3 (87.1, 87.6) | | 87.4 (87.2, 87.6) | | 87.4 (87.2, 87.6) | | 87.4 (87.2, 87.6) | | 87.4 (87.1, 87.6) | | 87.3 (87.1, 87.5) | |
| Low – Low | 88.6 (88.5, 88.6) | | 88.7 (88.6, 88.8) | | 88.7 (88.6, 88.7) | | 88.7 (88.6, 88.7) | | 88.7 (88.6, 88.8) | | 88.6 (88.5, 88.7) | |
| **Tangible-Belonging support** | |  | |  | |  | |  | |  | |  |
| High – High | 86.9 (86.7, 87.1) | | 87 (86.8, 87.1) | | 87 (86.8, 87.1) | | 87 (86.8, 87.1) | | 86.9 (86.7, 87.2) | | 86.9 (86.8, 87.0) | |
| High – Low | 88.5 (88.2, 88.7) | | 88.5 (88.3, 88.7) | | 88.5 (88.3, 88.7) | | 88.5 (88.3, 88.7) | | 88.6 (88.3, 88.9) | | 88.4 (88.2, 88.5) | |
| Low – High | 87.7 (87.5, 88) | | 87.8 (87.6, 88) | | 87.8 (87.6, 88) | | 87.8 (87.6, 88) | | 87.8 (87.5, 88.1) | | 87.6 (87.4, 87.7) | |
| Low – Low | 88.5 (88.4, 88.6) | | 88.6 (88.6, 88.7) | | 88.6 (88.6, 88.7) | | 88.6 (88.6, 88.7) | | 88.6 (88.5, 88.7) | | 88.6 (88.5, 88.6) | |
| **Belonging-Emotional support** | |  | |  | |  | |  | |  | |  |
| High – High | 86.9 (86.8, 87.1) | | 87.0 (86.9, 87.2) | | 87.0 (86.9, 87.2) | | 87.0 (86.9, 87.2) | | 87 (86.8, 87.2) | | 86.9 (86.8, 87.0) | |
| High – Low | 87.7 (87.5, 88) | | 87.8 (87.6, 88.1) | | 87.8 (87.6, 88.0) | | 87.8 (87.6, 88.0) | | 87.8 (87.5, 88.1) | | 87.7 (87.5, 87.9) | |
| Low – High | 87.4 (87.0, 87.7) | | 87.4 (87.2, 87.7) | | 87.4 (87.1, 87.6) | | 87.4 (87.1, 87.6) | | 87.5 (87.1, 87.8) | | 87.4 (87.1, 87.6) | |
| Low – Low | 88.6 (88.5, 88.7) | | 88.7 (88.6, 88.8) | | 88.7 (88.6, 88.8) | | 88.7 (88.6, 88.8) | | 88.7 (88.6, 88.8) | | 88.7 (88.6, 88.7) | |

Model A is the final main model (age, age2, education, smoking, province) plus health behaviours (alcohol, diet, physical activity and sleep).

Model B is the final main model (age, age2, education, smoking, province) plus psychological factors

Model C is the final main model (age, age2, education, smoking, province) plus marital status

Model D is the final main model (age, age2, education, smoking, province) plus chronic conditions

Model E is the final main model (age, age2, education, smoking, province) plus blood pressure (systolic blood pressure and diastolic blood pressure)

Model F is the final main model (age, age2, education, smoking, province) plus women’s reproductive status (parity, menopause status, hormone replacement therapy)

**S12 Table. Sensitive analysis of joint associations between functional social ties and adjusted mean WC in older men in CLSA (2011-15).**

|  | **Model A: final + behaviours** | **Model B: final + psychological** | **Model C: final + marital status** | **Model D: final + chronic disease** | **Model E: final + blood pressure** |
| --- | --- | --- | --- | --- | --- |
| **Informational support** | |  |  |  |  |
| High (20) | 99.7 (99.5, 99.8) | 99.8 (99.7, 99.9) | 99.75 (99.65, 99.85) | 99.75 (99.65, 99.85) | 99.75 (99.62, 99.89) |
| Low (4-19) | 100.3 (100.2, 100.4) | 100.3 (100.3, 100.4) | 100.4 (100.3, 100.4) | 100.4 (100.3, 100.4) | 100.3 (100.3, 100.4) |
| **Tangible support** | |  |  |  |  |
| High (20) | 100.0 (99.9, 100.1) | 100.1 (100, 100.1) | 100.1 (100, 100.1) | 100.1 (100, 100.1 | 100.1 (100, 100.1 |
| Low (4-19) | 100.2 (100.2, 100.3) | 100.2 (100.2, 100.3) | 100.2 (100.2, 100.3) | 100.2 (100.2, 100.3) | 100.2 (100.2, 100.3) |
| **Emotional support** | |  |  |  |  |
| High (35) | 100.0 (99.8, 100.1) | 100.1 (100, 100.2) | 100.1 (100, 100.2) | 100.1 (100, 100.2) | 100.1 (100, 100.2) |
| Low (7-34) | 100.2 (100.1, 100.3) | 100.2 (100.2, 100.3) | 100.2 (100.2, 100.3) | 100.2 (100.2, 100.3) | 100.2 (100.2, 100.3) |
| **Belonging support** | |  |  |  |  |
| High (20) | 100.1 (100, 100.2) | 100.2 (100.1, 100.3) | 100.2 (100.1, 100.3) | 100.2 (100.1, 100.3) | 100.2 (100.1, 100.3) |
| Low (4-19) | 100.2 (100.1, 100.2) | 100.2 (100.1, 100.2) | 100.2 (100.1, 100.3) | 100.2 (100.1, 100.3) | 100.2 (100.1, 100.3) |
| **Informational-Tangible support** | |  |  |  |  |
| High – High | 99.6 (99.5, 99.8) | 99.8 (99.6, 99.9) | 99.7 (99.6, 99.9) | 99.7 (99.6, 99.9) | 99.7 (99.6, 99.9) |
| High – Low | 99.8 (99.5, 100) | 99.8 (99.6, 100) | 99.8 (99.6, 100.0) | 99.8 (99.6, 100.0) | 99.8 (99.6, 100.1) |
| Low – High | 100.4 (100.2, 100.5) | 100.4 (100.3, 100.6) | 100.4 (100.3, 100.6) | 100.4 (100.3, 100.6) | 100.4 (100.3, 100.6) |
| Low – Low | 100.3 (100.2, 100.4) | 100.3 (100.2, 100.4) | 100.3 (100.3, 100.4) | 100.3 (100.3, 100.4) | 100.3 (100.2, 100.4) |
| **Informational-Emotional support** | |  |  |  |  |
| High – High | 99.7 (99.5, 99.8) | 99.8 (99.7, 99.9) | 99.77 (99.65, 99.89) | 99.77 (99.65, 99.89) | 99.77 (99.61, 99.93) |
| High – Low | 99.6 (99.4, 99.9) | 99.7 (99.5, 99.9) | 99.69 (99.5, 99.89) | 99.69 (99.5, 99.89) | 99.7 (99.44, 99.96) |
| Low – High | 100.9 (100.7, 101.2) | 101 (100.8, 101.2) | 101.0 (100.8, 101.2) | 101.0 (100.8, 101.2) | 101.0 (100.8, 101.3) |
| Low – Low | 100.3 (100.2, 100.3) | 100.3 (100.2, 100.3) | 100.3 (100.2, 100.4) | 100.3 (100.2, 100.4) | 100.3 (100.2, 100.4) |
| **Informational-Belonging support** | |  |  |  |  |
| High – High | 99.8 (99.6, 99.9) | 99.8 (99.7, 99.9) | 99.8 (99.7, 99.9) | 99.8 (99.7, 99.9) | 99.8 (99.7, 100.0) |
| High – Low | 99.4 (99.1, 99.6) | 99.6 (99.3, 99.8) | 99.6 (99.3, 99.8) | 99.6 (99.3, 99.8) | 99.6 (99.3, 99.9) |
| Low – High | 100.9 (100.7, 101.2) | 101 (100.8, 101.2) | 101 (100.8, 101.2) | 101 (100.8, 101.2) | 101 (100.8, 101.2) |
| Low – Low | 100.2 (100.2, 100.3) | 100.2 (100.2, 100.3) | 100.3 (100.2, 100.3) | 100.3 (100.2, 100.3) | 100.3 (100.2, 100.3) |
| **Tangible-Emotional support** | |  |  |  |  |
| High – High | 99.8 (99.7, 100.0) | 99.9 (99.8, 100.0) | 99.9 (99.8, 100.0) | 99.9 (99.8, 100.0) | 99.9 (99.7, 100.1) |
| High – Low | 100.1 (100.0, 100.3) | 100.2 (100.1, 100.4) | 100.2 (100.1, 100.3) | 100.2 (100.1, 100.3) | 100.2 (100.1, 100.4) |
| Low – High | 100.4 (100.2, 100.7) | 100.6 (100.4, 100.8) | 100.5 (100.3, 100.7) | 100.5 (100.3, 100.7) | 100.6 (100.3, 100.8) |
| Low – Low | 100.2 (100.1, 100.3) | 100.2 (100.1, 100.3) | 100.2 (100.2, 100.3) | 100.2 (100.2, 100.3) | 100.2 (100.1, 100.3) |
| **Tangible-Belonging support** | |  |  |  |  |
| High – High | 99.9 (99.8, 100.0) | 99.98 (99.86, 100.1) | 100 (99.9, 100.1) | 100 (99.9, 100.1) | 100.0 (99.8, 100.1) |
| High – Low | 100.1 (99.9, 100.2) | 100.2 (100.0, 100.3) | 100.2 (100.0, 100.3) | 100.2 (100.0, 100.3) | 100.2 (100, 100.4) |
| Low – High | 100.7 (100.5, 101) | 100.7 (100.6, 100.9) | 100.8 (100.6, 100.9) | 100.8 (100.6, 100.9) | 100.8 (100.5, 101) |
| Low – Low | 100.2 (100.1, 100.3) | 100.2 (100.1, 100.3) | 100.2 (100.1, 100.3) | 100.2 (100.1, 100.3) | 100.2 (100.1, 100.3) |
| **Belonging-Emotional support** | |  |  |  |  |
| High – High | 99.9 (99.8, 100.1) | 100 (99.9, 100.1) | 100.0 (99.9, 100.1) | 100.0 (99.9, 100.1) | 100.0 (99.8, 100.2) |
| High – Low | 100.6 (100.4, 100.8) | 100.6 (100.4, 100.8) | 100.6 (100.4, 100.8) | 100.6 (100.4, 100.8) | 100.6 (100.4, 100.8) |
| Low – High | 100.2 (99.9, 100.5) | 100.4 (100.1, 100.6) | 100.4 (100.1, 100.6) | 100.4 (100.1, 100.6) | 100.3 (100.0, 100.6) |
| Low – Low | 100.2 (100.1, 100.2) | 100.2 (100.1, 100.2) | 100.2 (100.1, 100.3) | 100.2 (100.1, 100.3) | 100.2 (100.1, 100.3) |

Model A is the final main model (age, age2, education, smoking, province) plus health behaviours (alcohol, diet, physical activity and sleep)

Model B is the final main model (age, age2, education, smoking, province) plus psychological factors

Model C is the final main model (age, age2, education, smoking, province) plus marital status

Model D is the final main model (age, age2, education, smoking, province) plus chronic conditions

Model E is the final main model (age, age2, education, smoking, province) plus blood pressure (systolic blood pressure and diastolic blood pressure).

**S13 Table. Sensitivity analysis of joint associations between functional social ties and mean SBP in older women in CLSA (2011-15).**

|  | **Model A: final + behaviours** | **Model B: final + psychological** | **Model C: final + marital status** | **Model D: final + chronic disease** | **Model F: final + BMI, WC** | **Model G: final + reproductive** |
| --- | --- | --- | --- | --- | --- | --- |
| **Informational support** | |  |  |  |  |  |
| High (20) | 118.4 (118.2, 118.6) | 118.4 (118.2, 118.6) | 118.5 (118.3, 118.7) | 118.5 (118.3, 118.7) | 118.5 (118.3, 118.8) | 118.7 (118.5, 118.9) |
| Low (4-19) | 119.8 (119.7, 120.0) | 119.8 (119.7, 120.0) | 120.0 (119.8, 120.1) | 120.0 (119.8, 120.1) | 120.0 (119.8, 120.1) | 120.2 (120.1, 120.4) |
| **Tangible support** | |  |  |  |  |  |
| High (20) | 119.1 (118.8, 119.3) | 119.1 (118.8, 119.3) | 119.1 (118.9, 119.3) | 119.1 (118.9, 119.3) | 119.1 (118.8, 119.3) | 119.3 (119.1, 119.5) |
| Low (4-19) | 119.6 (119.5, 119.7) | 119.7 (119.6, 119.9) | 119.7 (119.6, 119.9) | 119.7 (119.6, 119.9) | 119.7 (119.6, 119.9) | 120.0 (119.9, 120.1) |
| **Emotional support** | |  |  |  |  |  |
| High (35) | 118.5 (118.3, 118.8) | 118.6 (118.4, 118.8) | 118.6 (118.4, 118.8) | 118.6 (118.4, 118.8) | 118.6 (118.3, 118.9) | 118.8 (118.6, 119.1) |
| Low (7-34) | 119.7 (119.6, 119.9) | 119.9 (119.7, 120.0) | 119.9 (119.8, 120) | 119.9 (119.8, 120) | 119.9 (119.7, 120.0) | 120.1 (120.0, 120.3) |
| **Belonging support** | |  |  |  |  |  |
| High (20) | 118.8 (118.6, 119.0) | 118.9 (118.7, 119.1) | 118.9 (118.7, 119.1) | 118.9 (118.7, 119.1) | 118.9 (118.7, 119.2) | 119.2 (119.0, 119.5) |
| Low (4-19) | 119.7 (119.5, 119.8) | 119.8 (119.7, 119.9) | 119.8 (119.7, 119.9) | 119.8 (119.7, 119.9) | 119.8 (119.6, 120.0) | 120.0 (119.9, 120.2) |
| **Informational-Tangible support** | |  |  |  |  |  |
| High – High | 118.5 (118.3, 118.8) | 118.6 (118.3, 118.8) | 118.6 (118.3, 118.8) | 118.6 (118.3, 118.8) | 118.5 (118.2, 118.8) | 118.7 (118.5, 119) |
| High – Low | 118.2 (117.9, 118.5) | 118.5 (118.2, 118.9) | 118.5 (118.2, 118.9) | 118.5 (118.2, 118.9) | 118.5 (118.1, 119) | 118.6 (118.3, 119) |
| Low – High | 120 (119.7, 120.3) | 119.9 (119.6, 120.3) | 120.0 (119.6, 120.3) | 120.0 (119.6, 120.3) | 120.1 (119.6, 120.5) | 120.3 (119.9, 120.6) |
| Low – Low | 119.8 (119.7, 120.0) | 119.9 (119.8, 120.1) | 120.0 (119.8, 120.1) | 120.0 (119.8, 120.1) | 119.9 (119.8, 120.1) | 120.2 (120.1, 120.4) |
| **Informational-Emotional support** | |  |  |  |  |  |
| High – High | 118.1 (117.9, 118.4) | 118.2 (118, 118.4) | 118.2 (118, 118.4) | 118.2 (118, 118.4) | 118.2 (117.9, 118.5) | 118.4 (118.1, 118.6) |
| High – Low | 119.1 (118.7, 119.4) | 119.4 (119, 119.7) | 119.3 (119, 119.7) | 119.3 (119, 119.7) | 119.3 (118.9, 119.8) | 119.5 (119.1, 119.9) |
| Low – High | 120.1 (119.6, 120.7) | 120.1 (119.5, 120.6) | 120.1 (119.6, 120.6) | 120.1 (119.6, 120.6) | 120.2 (119.5, 120.8) | 120.5 (120, 121.0) |
| Low – Low | 119.8 (119.7, 119.9) | 119.9 (119.8, 120.1) | 119.9 (119.8, 120.1) | 119.9 (119.8, 120.1) | 119.9 (119.8, 120.1) | 120.2 (120.1, 120.4) |
| **Informational-Belonging support** | |  |  |  |  |  |
| High – High | 118.4 (118.2, 118.7) | 118.5 (118.3, 118.8) | 118.5 (118.3, 118.8) | 118.5 (118.3, 118.8) | 118.5 (118.2, 118.8) | 118.8 (118.6, 119.0) |
| High – Low | 118.4 (118, 118.8) | 118.5 (118.1, 118.9) | 118.5 (118.2, 118.9) | 118.5 (118.2, 118.9) | 118.6 (118.1, 119.1) | 118.5 (118.1, 118.9) |
| Low – High | 119.9 (119.5, 120.3) | 119.9 (119.5, 120.3) | 120.0 (119.5, 120.4) | 120.0 (119.5, 120.4) | 120 (119.5, 120.5) | 120.5 (120.1, 120.9) |
| Low – Low | 119.8 (119.7, 120.0) | 119.9 (119.8, 120.1) | 120.0 (119.8, 120.1) | 120.0 (119.8, 120.1) | 120.0 (119.8, 120.1) | 120.2 (120.1, 120.3) |
| **Tangible-Emotional support** | |  |  |  |  |  |
| High – High | 118.5 (118.2, 118.8) | 118.5 (118.2, 118.8) | 118.5 (118.2, 118.8) | 118.5 (118.2, 118.8) | 118.6 (118.2, 118.9) | 118.7 (118.5, 119) |
| High – Low | 119.9 (119.5, 120.2) | 119.9 (119.5, 120.2) | 119.9 (119.6, 120.2) | 119.9 (119.6, 120.2) | 119.8 (119.4, 120.2) | 120.1 (119.8, 120.4) |
| Low – High | 118.6 (118.2, 119) | 118.7 (118.4, 119.1) | 118.8 (118.4, 119.1) | 118.8 (118.4, 119.1) | 118.7 (118.2, 119.2) | 119 (118.6, 119.4) |
| Low – Low | 119.7 (119.6, 119.8) | 119.9 (119.7, 120) | 119.9 (119.7, 120.0) | 119.9 (119.7, 120.0) | 119.9 (119.7, 120.1) | 120.1 (120, 120.3) |
| **Tangible-Belonging support** | |  |  |  |  |  |
| High – High | 118.5 (118.2, 118.7) | **118.5 (118.2, 118.7)** | 118.5 (118.2, 118.7) | 118.5 (118.2, 118.7) | 118.5 (118.2, 118.8) | 118.7 (118.5, 119) |
| High – Low | 120.1 (119.8, 120.5) | **120.1 (119.8, 120.5)** | 120.1 (119.8, 120.5) | 120.1 (119.8, 120.5) | 120.2 (119.7, 120.6) | 120.3 (120, 120.7) |
| Low – High | 119.4 (119.1, 119.8) | **119.7 (119.3, 120.0)** | 119.7 (119.3, 120) | 119.7 (119.3, 120) | 119.7 (119.2, 120.1) | 120.2 (119.8, 120.5) |
| Low – Low | 119.6 (119.5, 119.7) | **119.7 (119.6, 119.9)** | 119.8 (119.6, 119.9) | 119.8 (119.6, 119.9) | 119.8 (119.6, 119.9) | 120 (119.8, 120.1) |
| **Belonging-Emotional support** | |  |  |  |  |  |
| High – High | 118.5 (118.2, 118.7) | 118.5 (118.3, 118.8) | 118.5 (118.3, 118.8) | 118.5 (118.3, 118.8) | 118.6 (118.2, 118.9) | 118.8 (118.6, 119.1) |
| High – Low | 119.6 (119.3, 120) | 119.8 (119.4, 120.2) | 119.8 (119.4, 120.1) | 119.8 (119.4, 120.1) | 119.7 (119.3, 120.2) | 120.2 (119.9, 120.6) |
| Low – High | 118.7 (118.3, 119.2) | 118.8 (118.3, 119.2) | 118.8 (118.3, 119.2) | 118.8 (118.3, 119.2) | 118.8 (118.2, 119.4) | 118.8 (118.3, 119.3) |
| Low – Low | 119.7 (119.6, 119.9) | 119.9 (119.7, 120) | 119.9 (119.8, 120.0) | 119.9 (119.8, 120.0) | 119.9 (119.7, 120.1) | 120.1 (120, 120.3) |

Model A is the final main model (age, age2, education, smoking, province) plus health behaviours (alcohol, diet, physical activity and sleep)

Model B is the final main model (age, age2, education, smoking, province) plus psychological factors

Model C is the final main model (age, age2, education, smoking, province) plus marital status

Model D is the final main model (age, age2, education, smoking, province) plus chronic conditions

Model E is the final main model (age, age2, education, smoking, province) plus anthropometry (body mass index, waist circumference)

Model F is the final main model (age, age2, education, smoking, province) plus women’s reproductive status (parity, menopause status, hormone replacement therapy)

**S14 Table. Sensitivity analysis of joint associations between functional social ties and adjusted mean SBP in older men in CLSA (2011-15).**

|  | **Model A: final + behaviours** | **Model B: final + psychological** | **Model C: final + marital status** | **Model D: final + chronic disease** | **Model E: final + BMI, WC** |
| --- | --- | --- | --- | --- | --- |
| **Informational support** | |  |  |  |  |
| High (20) | 121.9 (121.8, 122.1) | 122 (121.9, 122.1) | 122 (121.9, 122.1) | 122 (121.9, 122.1) | 122 (121.8, 122.2) |
| Low (4-19) | 122.5 (122.5, 122.6) | 122.6 (122.5, 122.7) | 122.6 (122.5, 122.7) | 122.6 (122.5, 122.7) | 122.6 (122.5, 122.7) |
| **Tangible support** | |  |  |  |  |
| High (20) | 121.9 (121.8, 122) | 122 (121.9, 122.1) | 122 (121.9, 122.1) | 122 (121.9, 122.1) | 122 (121.8, 122.1) |
| Low (4-19) | 122.6 (122.6, 122.7) | 122.7 (122.6, 122.8) | 122.7 (122.6, 122.8) | 122.7 (122.6, 122.8) | 122.7 (122.6, 122.8) |
| **Emotional support** | |  |  |  |  |
| High (35) | 121.9 (121.7, 122) | 121.9 (121.8, 122.0) | 121.9 (121.8, 122.0) | 121.9 (121.8, 122.0) | 121.9 (121.7, 122.1) |
| Low (7-34) | 122.6 (122.5, 122.6) | 122.6 (122.6, 122.7) | 122.6 (122.6, 122.7) | 122.6 (122.6, 122.7) | 122.6 (122.5, 122.7) |
| **Belonging support** | |  |  |  |  |
| High (20) | 122.3 (122.2, 122.4) | 122.3 (122.2, 122.4) | 122.3 (122.2, 122.4) | 122.3 (122.2, 122.4) | 122.3 (122.2, 122.5) |
| Low (4-19) | 122.4 (122.3, 122.5) | 122.5 (122.4, 122.6) | 122.5 (122.4, 122.6) | 122.5 (122.4, 122.6) | 122.5 (122.4, 122.6) |
| **Informational-Tangible support** | |  |  |  |  |
| High – High | 121.8 (121.7, 121.9) | 121.9 (121.7, 122.0) | 121.9 (121.7, 122.0) | 121.9 (121.7, 122.0) | 121.9 (121.7, 122.1) |
| High – Low | 122.3 (122.0, 122.5) | 122.3 (122.1, 122.6) | 122.3 (122.1, 122.6) | 122.3 (122.1, 122.6) | 122.4 (122.0, 122.7) |
| Low – High | 122 (121.8, 122.2) | 122.1 (122, 122.3) | 122.1 (122, 122.3) | 122.1 (122, 122.3) | 122.1 (121.9, 122.4) |
| Low – Low | 122.7 (122.7, 122.8) | 122.7 (122.6, 122.8) | 122.7 (122.7, 122.8) | 122.7 (122.7, 122.8) | 122.7 (122.6, 122.8) |
| **Informational-Emotional support** | |  |  |  |  |
| High – High | 121.8 (121.7, 122) | 121.9 (121.7, 122.0) | 121.9 (121.7, 122.0) | 121.9 (121.7, 122.0) | 121.8 (121.6, 122.0) |
| High – Low | 122.2 (122, 122.5) | 122.3 (122.1, 122.6) | 122.3 (122.1, 122.6) | 122.3 (122.1, 122.6) | 122.5 (122.1, 122.8) |
| Low – High | 122 (121.7, 122.2) | 122 (121.7, 122.2) | 122 (121.7, 122.2) | 122 (121.7, 122.2) | 122.1 (121.8, 122.5) |
| Low – Low | 122.6 (122.5, 122.7) | 122.7 (122.6, 122.7) | 122.7 (122.6, 122.7) | 122.7 (122.6, 122.7) | 122.7 (122.5, 122.8) |
| **Informational-Belonging support** | |  |  |  |  |
| High – High | 122.0 (121.9, 122.2) | 122.1 (121.9, 122.2) | 122.1 (122, 122.2) | 122.1 (122, 122.2) | 122.1 (121.9, 122.3) |
| High – Low | 121.6 (121.3, 121.9) | 121.7 (121.4, 121.9) | 121.7 (121.4, 121.9) | 121.7 (121.4, 121.9) | 121.5 (121.2, 121.9) |
| Low – High | 122.8 (122.6, 123.0) | 122.8 (122.6, 123.0) | 122.8 (122.6, 123.1) | 122.8 (122.6, 123.1) | 122.8 (122.5, 123.1) |
| Low – Low | 122.5 (122.4, 122.6) | 122.6 (122.5, 122.6) | 122.6 (122.5, 122.7) | 122.6 (122.5, 122.7) | 122.6 (122.5, 122.7) |
| **Tangible-Emotional support** | |  |  |  |  |
| High – High | 121.7 (121.6, 121.9) | 121.8 (121.7, 122) | 121.8 (121.7, 121.9) | 121.8 (121.7, 121.9) | 121.8 (121.6, 122.0) |
| High – Low | 122.1 (121.9, 122.2) | 122.2 (122.1, 122.4) | 122.2 (122.0, 122.4) | 122.2 (122.0, 122.4) | 122.2 (122, 122.4) |
| Low – High | 122.2 (122, 122.5) | 122.2 (121.9, 122.4) | 122.2 (122, 122.5) | 122.2 (122, 122.5) | 122.1 (121.8, 122.5) |
| Low – Low | 122.7 (122.6, 122.8) | 122.7 (122.7, 122.8) | 122.8 (122.7, 122.8) | 122.8 (122.7, 122.8) | 122.8 (122.6, 122.9) |
| **Tangible-Belonging support** | |  |  |  |  |
| High – High | 121.9 (121.8, 122.1) | 122 (121.9, 122.1) | 122.0 (121.9, 122.1) | 122.0 (121.9, 122.1) | 122.0 (121.8, 122.2) |
| High – Low | 121.8 (121.6, 122) | 122 (121.8, 122.1) | 121.9 (121.8, 122.1) | 121.9 (121.8, 122.1) | 121.9 (121.7, 122.2) |
| Low – High | 123.2 (122.9, 123.4) | 123.2 (122.9, 123.4) | 123.2 (123, 123.4) | 123.2 (123, 123.4) | 123.2 (122.9, 123.5) |
| Low – Low | 122.6 (122.5, 122.7) | 122.6 (122.5, 122.7) | 122.6 (122.6, 122.7) | 122.6 (122.6, 122.7) | 122.6 (122.5, 122.7) |
| **Belonging-Emotional support** | |  |  |  |  |
| High – High | 122 (121.8, 122.1) | 122.0 (121.9, 122.2) | 122.0 (121.9, 122.2) | 122.0 (121.9, 122.2) | 122.0 (121.8, 122.2) |
| High – Low | 122.9 (122.7, 123.1) | 123 (122.7, 123.2) | 123 (122.8, 123.2) | 123 (122.8, 123.2) | 123.0 (122.7, 123.3) |
| Low – High | 121.4 (121.1, 121.7) | 121.4 (121.1, 121.7) | 121.4 (121.1, 121.7) | 121.4 (121.1, 121.7) | 121.4 (121.0, 121.8) |
| Low – Low | 122.5 (122.4, 122.6) | 122.6 (122.5, 122.7) | 122.6 (122.5, 122.7) | 122.6 (122.5, 122.7) | 122.6 (122.5, 122.7) |

Model A is the final main model (age, age2, education, smoking, province) plus health behaviours (alcohol, diet, physical activity and sleep)

Model B is the final main model (age, age2, education, smoking, province) plus psychological factors

Model C is the final main model (age, age2, education, smoking, province) plus marital status

Model D is the final main model (age, age2, education, smoking, province) plus chronic conditions

Model E is the final main model (age, age2, education, smoking, province) plus anthropometry (body mass index, waist circumference).

**S15 Table. Sensitivity analysis of joint associations between functional social ties and adjusted mean DBP in older women in CLSA (2011-15).**

|  | **Model A: final + behaviours** | **Model B: final + psychological** | **Model C: final + marital status** | **Model D: final + chronic disease** | **Model E: final + BMI, WC** | **Model F: final + reproductive** |
| --- | --- | --- | --- | --- | --- | --- |
| **Informational support** | |  |  |  |  |  |
| High (20) | 71.9 (71.9, 72.0) | 71.9 (71.9, 72.0) | 72.0 (71.9, 72.0) | 72.0 (71.9, 72.0) | 72.0 (71.9, 72.1) | 71.9 (71.8, 71.9) |
| Low (4-19) | 71.6 (71.6, 71.6) | 71.6 (71.6, 71.6) | 71.6 (71.6, 71.7) | 71.6 (71.6, 71.7) | 71.6 (71.6, 71.7) | 71.6 (71.6, 71.7) |
| **Tangible support** |  |  |  |  |  |  |
| High (20) | 71.7 (71.6, 71.7) | 71.7 (71.6, 71.7) | 71.7 (71.6, 71.7) | 71.7 (71.6, 71.7) | 71.7 (71.6, 71.8) | 71.6 (71.5, 71.6) |
| Low (4-19) | 71.7 (71.7, 71.7) | 71.8 (71.7, 71.8) | 71.8 (71.7, 71.8) | 71.8 (71.7, 71.8) | 71.8 (71.7, 71.8) | 71.7 (71.7, 71.8) |
| **Emotional support** |  |  |  |  |  |  |
| High (35) | 72.0 (71.9, 72.0) | 72.0 (71.9, 72.0) | 72.0 (71.9, 72.0) | 72.0 (71.9, 72.0) | 72.0 (71.9, 72.1) | 71.9 (71.9, 72.0) |
| Low (7-34) | 71.6 (71.6, 71.6) | 71.7 (71.6, 71.7) | 71.6 (71.6, 71.7) | 71.6 (71.6, 71.7) | 71.6 (71.6, 71.7) | 71.6 (71.6, 71.6) |
| **Belonging support** |  |  |  |  |  |  |
| High (20) | 71.9 (71.8, 71.9) | 71.9 (71.9, 72.0) | 71.9 (71.9, 72.0) | 71.9 (71.9, 72.0) | 71.9 (71.8, 72.0) | 71.9 (71.8, 72.0) |
| Low (4-19) | 71.6 (71.6, 71.7) | 71.7 (71.6, 71.7) | 71.7 (71.6, 71.7) | 71.7 (71.6, 71.7) | 71.7 (71.6, 71.7) | 71.6 (71.6, 71.6) |
| **Informational-Tangible support** | |  |  |  |  |  |
| High – High | 71.9 (71.8, 71.9) | 71.9 (71.8, 71.9) | 71.8 (71.8, 71.9) | 71.8 (71.8, 71.9) | 71.8 (71.7, 71.9) | 71.7 (71.7, 71.8) |
| High – Low | 72.0 (72.0, 72.1) | 72.2 (72.1, 72.3) | 72.2 (72.1, 72.3) | 72.2 (72.1, 72.3) | 72.2 (72.0, 72.3) | 72.1 (72.0, 72.2) |
| Low – High | 71.4 (71.3, 71.5) | 71.3 (71.2, 71.4) | 71.3 (71.2, 71.4) | 71.3 (71.2, 71.4) | 71.4 (71.2, 71.5) | 71.3 (71.2, 71.4) |
| Low – Low | 71.6 (71.6, 71.7) | 71.7 (71.6, 71.7) | 71.7 (71.6, 71.7) | 71.7 (71.6, 71.7) | 71.7 (71.6, 71.7) | 71.7 (71.6, 71.7) |
| **Informational-Emotional support** | |  |  |  |  |  |
| High – High | 71.9 (71.8, 72.0) | 71.9 (71.9, 72.0) | 71.9 (71.9, 72.0) | 71.9 (71.9, 72.0) | 71.9 (71.8, 72.0) | 71.8 (71.8, 71.9) |
| High – Low | 72.0 (71.9, 72.1) | 72.1 (72.0, 72.2) | 72.1 (72.0, 72.2) | 72.1 (72.0, 72.2) | 72.1 (72.0, 72.3) | 72.0 (71.8, 72.1) |
| Low – High | 72.3 (72.1, 72.4) | 72.2 (72.1, 72.3) | 72.2 (72.1, 72.4) | 72.2 (72.1, 72.4) | 72.3 (72.1, 72.5) | 72.5 (72.3, 72.6) |
| Low – Low | 71.5 (71.5, 71.6) | 71.6 (71.6, 71.6) | 71.6 (71.6, 71.6) | 71.6 (71.6, 71.6) | 71.6 (71.5, 71.6) | 71.5 (71.5, 71.6) |
| **Informational-Belonging support** | |  |  |  |  |  |
| High – High | 71.9 (71.8, 71.9) | 71.9 (71.9, 72.0) | 71.9 (71.8, 72.0) | 71.9 (71.8, 72.0) | 71.9 (71.8, 72.0) | 71.8 (71.8, 71.9) |
| High – Low | 72.1 (72.0, 72.2) | 72.2 (72.1, 72.3) | 72.2 (72.0, 72.3) | 72.2 (72.0, 72.3) | 72.1 (72.0, 72.3) | 71.9 (71.8, 72.0) |
| Low – High | 72.0 (71.9, 72.1) | 72.0 (71.9, 72.1) | 72.0 (71.9, 72.1) | 72.0 (71.9, 72.1) | 72.0 (71.8, 72.1) | 72.1 (72.0, 72.2) |
| Low – Low | 71.6 (71.5, 71.6) | 71.6 (71.6, 71.6) | 71.6 (71.6, 71.6) | 71.6 (71.6, 71.6) | 71.6 (71.5, 71.7) | 71.6 (71.5, 71.6) |
| **Tangible-Emotional support** | |  |  |  |  |  |
| High – High | 71.8 (71.8, 71.9) | 71.8 (71.8, 71.9) | 71.8 (71.8, 71.9) | 71.8 (71.8, 71.9) | 71.8 (71.7, 71.9) | 71.7 (71.6, 71.8) |
| High – Low | 71.5 (71.4, 71.6) | 71.4 (71.3, 71.5) | 71.4 (71.3, 71.5) | 71.4 (71.3, 71.5) | 71.4 (71.3, 71.6) | 71.3 (71.2, 71.4) |
| Low – High | 72.2 (72.1, 72.3) | 72.3 (72.2, 72.3) | 72.2 (72.2, 72.3) | 72.2 (72.2, 72.3) | 72.2 (72.1, 72.4) | 72.3 (72.2, 72.4) |
| Low – Low | 71.6 (71.6, 71.7) | 71.7 (71.6, 71.7) | 71.7 (71.6, 71.7) | 71.7 (71.6, 71.7) | 71.7 (71.6, 71.7) | 71.6 (71.6, 71.7) |
| **Tangible-Belonging support** |  |  |  |  |  |  |
| High – High | 71.9 (71.8, 71.9) | 71.8 (71.8, 71.9) | 71.8 (71.8, 71.9) | 71.8 (71.8, 71.9) | 71.8 (71.7, 71.9) | 71.7 (71.6, 71.8) |
| High – Low | 71.3 (71.2, 71.4) | 71.4 (71.3, 71.5) | 71.3 (71.2, 71.4) | 71.3 (71.2, 71.4) | 71.4 (71.2, 71.5) | 71.3 (71.2, 71.4) |
| Low – High | 72.0 (71.9, 72.0) | 72.1 (72.0, 72.2) | 72.1 (72.0, 72.3) | 72.1 (72.0, 72.2) | 72.1 (71.92, 72.2) | 72.2 (72.1, 72.3) |
| Low – Low | 71.7 (71.6, 71.7) | 71.7 (71.7, 71.7) | 71.7 (71.7, 71.7) | 71.7 (71.7, 71.7) | 71.7 (71.6, 71.8) | 71.6 (71.6, 71.7) |
| **Belonging-Emotional support** |  |  |  |  |  |  |
| High – High | 71.9 (71.8, 72.0) | 71.9 (71.9, 72.0) | 71.9 (71.9, 72.0) | 71.9 (71.9, 72.0) | 71.9 (71.8, 72.0) | 71.9 (71.8, 72.0) |
| High – Low | 71.9 (71.8, 72.0) | 71.9 (71.8, 72.0) | 71.9 (71.8, 72.0) | 71.9 (71.8, 72.0) | 71.9 (71.8, 72.1) | 71.9 (71.8, 72.0) |
| Low – High | 72.2 (72.1, 72.4) | 72.2 (72.1, 72.3) | 72.2 (72.1, 72.3) | 72.2 (72.1, 72.3) | 72.2 (72.0, 72.4) | 72.1 (72.0, 72.2) |
| Low – Low | 71.6 (71.5, 71.6) | 71.6 (71.6, 71.7) | 71.6 (71.6, 71.7) | 71.6 (71.6, 71.7) | 71.6 (71.6, 71.7) | 71.6 (71.5, 71.6) |

Model A is the final main model (age, age2, education, smoking, province) plus health behaviours (alcohol, diet, physical activity and sleep)

Model B is the final main model (age, age2, education, smoking, province) plus psychological factors

Model C is the final main model (age, age2, education, smoking, province) plus marital status

Model D is the final main model (age, age2, education, smoking, province) plus chronic conditions

Model E is the final main model (age, age2, education, smoking, province) plus anthropometry (body mass index, waist circumference)

Model F is the final main model (age, age2, education, smoking, province) plus women’s reproductive status (parity, menopause status, hormone replacement therapy)

**S16 Table. Sensitivity analysis of joint associations between functional social ties and adjusted mean DBP in older men in CLSA (2011-15).**

|  | **Model A: final + behaviours** | **Model B: final + psychological** | **Model C: final + marital status** | **Model D: final + chronic disease** | **Model F: final + BMI, WC** |
| --- | --- | --- | --- | --- | --- |
| **Informational support** |  |  |  |  |  |
| High (20) | 76.2 (76.1, 76.3) | 76.2 (76.1, 76.3) | 76.2 (76.1, 76.3) | 76.2 (76.1, 76.3) | 76.2 (76.1, 76.3) |
| Low (4-19) | 76.1 (76.0, 76.1) | 76.1 (76.0, 76.1) | 76.1 (76.0, 76.1) | 76.1 (76.0, 76.1) | 76.1 (76.0, 76.1) |
| **Tangible support** | |  |  |  |  |
| High (20) | 75.8 (75.7, 75.9) | 75.8 (75.7, 75.9) | 75.8 (75.7, 75.9) | 75.8 (75.7, 75.9) | 75.8 (75.7, 75.9) |
| Low (4-19) | 76.3 (76.2, 76.3) | 76.3 (76.2, 76.3) | 76.3 (76.2, 76.3) | 76.3 (76.2, 76.3) | 76.3 (76.2, 76.3) |
| **Emotional support** | |  |  |  |  |
| High (35) | 76.1 (76.0, 76.2) | 76.1 (76.0, 76.2) | 76.1 (76.0, 76.2) | 76.1 (76.0, 76.2) | 76.1 (75.9, 76.2) |
| Low (7-34) | 76.1 (76.0, 76.2) | 76.1 (76.0, 76.2) | 76.1 (76.0, 76.2) | 76.1 (76.0, 76.2) | 76.1 (76.0, 76.2) |
| **Belonging support** | |  |  |  |  |
| High (20) | 76.1 (76.0, 76.2) | 76.1 (76.0, 76.2) | 76.1 (76.0, 76.2) | 76.1 (76.0, 76.2) | 76.1 (76.0, 76.2) |
| Low (4-19) | 76.1 (76.0, 76.1) | 76.1 (76.0, 76.2) | 76.1 (76.0, 76.2) | 76.1 (76.0, 76.2) | 76.1 (76.0, 76.2) |
| **Informational-Tangible support** | |  |  |  |  |
| High – High | 76.0 (75.9, 76.1) | 76.0 (75.9, 76.2) | 76.0 (75.9, 76.1) | 76.0 (75.9, 76.1) | 76.0 (75.9, 76.1) |
| High – Low | 76.7 (76.5, 76.9) | 76.7 (76.5, 76.9) | 76.7 (76.5, 77.0) | 76.7 (76.5, 76.9) | 76.8 (76.5, 77.0) |
| Low – High | 75.5 (75.3, 75.6) | 75.5 (75.4, 75.6) | 75.5 (75.4, 75.6) | 75.5 (75.4, 75.6) | 75.5 (75.4, 75.7) |
| Low – Low | 76.2 (76.1, 76.3) | 76.2 (76.1, 76.3) | 76.2 (76.1, 76.3) | 76.2 (76.1, 76.3) | 76.2 (76.1, 76.3) |
| **Informational-Emotional support** | |  |  |  |  |
| High – High | 76.1 (76.0, 76.2) | 76.1 (76.0, 76.2) | 76.1 (76.0, 76.2) | 76.1 (76.0, 76.2) | 76.1 (75.9, 76.2) |
| High – Low | 76.5 (76.3, 76.7) | 76.5 (76.3, 76.8) | 76.6 (76.3, 76.8) | 76.6 (76.3, 76.8) | 76.6 (76.3, 76.8) |
| Low – High | 76.0 (75.8, 76.2) | 75.97 (75.7, 76.2) | 76.0 (75.7, 76.2) | 76.0 (75.7, 76.2) | 76.0 (75.8, 76.3) |
| Low – Low | 76.1 (76.0, 76.1) | 76.1 (76.0, 76.1) | 76.1 (76, 76.1) | 76.1 (76, 76.1) | 76.1 (76.0, 76.1) |
| **Informational-Belonging support** | |  |  |  |  |
| High – High | 76.1 (76.0, 76.2) | 76.1 (76, 76.2) | 76.1 (76, 76.2) | 76.1 (76, 76.2) | 76.1 (76.0, 76.3) |
| High – Low | 76.5 (76.2, 76.7) | 76.5 (76.3, 76.8) | 76.5 (76.3, 76.8) | 76.5 (76.3, 76.8) | 76.4 (76.2, 76.7) |
| Low – High | 76.2 (76.0, 76.3) | 76.1 (76.0, 76.3) | 76.1 (76.0, 76.3) | 76.1 (76.0, 76.3) | 76.1 (75.9, 76.3) |
| Low – Low | 76.0 (76.0, 76.1) | 76.0 (76.0, 76.1) | 76.1 (76.0, 76.1) | 76.1 (76.0, 76.1) | 76.1 (76.0, 76.1) |
| **Tangible-Emotional support** | |  |  |  |  |
| High – High | 75.9 (75.8, 76.1) | 75.9 (75.8, 76.1) | 75.9 (75.8, 76.0) | 75.9 (75.8, 76.0) | 75.9 (75.8, 76.1) |
| High – Low | 75.6 (75.5, 75.7) | 75.6 (75.5, 75.8) | 75.6 (75.5, 75.8) | 75.6 (75.5, 75.8) | 75.6 (75.5, 75.8) |
| Low – High | 76.5 (76.3, 76.7) | 76.5 (76.3, 76.71) | 76.5 (76.3, 76.7) | 76.5 (76.3, 76.7) | 76.5 (76.2, 76.7) |
| Low – Low | 76.2 (76.1, 76.3) | 76.2 (76.2, 76.3) | 76.2 (76.2, 76.3) | 76.2 (76.2, 76.3) | 76.2 (76.2, 76.3) |
| **Tangible-Belonging support** | |  |  |  |  |
| High – High | 76.0 (75.8, 76.1) | 76.0 (75.8, 76.1) | 76.0 (75.8, 76.1) | 76.0 (75.8, 76.1) | 76.0 (75.8, 76.1) |
| High – Low | 75.5 (75.3, 75.6) | 75.5 (75.4, 75.7) | 75.5 (75.4, 75.7) | 75.5 (75.4, 75.7) | 75.5 (75.3, 75.7) |
| Low – High | 76.6 (76.4, 76.8) | 76.6 (76.4, 76.8) | 76.6 (76.4, 76.8) | 76.6 (76.4, 76.8) | 76.6 (76.4, 76.8) |
| Low – Low | 76.2 (76.1, 76.3) | 76.2 (76.1, 76.3) | 76.2 (76.1, 76.3) | 76.2 (76.1, 76.3) | 76.2 (76.1, 76.3) |
| **Belonging-Emotional support** | |  |  |  |  |
| High – High | 76.0 (75.9, 76.2) | 76.0 (75.9, 76.2) | 76.0 (75.9, 76.2) | 76.0 (75.9, 76.2) | 76.0 (75.9, 76.16) |
| High – Low | 76.3 (76.2, 76.5) | 76.3 (76.1, 76.5) | 76.3 (76.2, 76.5) | 76.3 (76.2, 76.5) | 76.4 (76.2, 76.6) |
| Low – High | 76.2 (75.9, 76.4) | 76.2 (75.9, 76.4) | 76.2 (75.9, 76.4) | 76.2 (75.9, 76.4) | 76.2 (75.9, 76.5) |
| Low – Low | 76.1 (76.0, 76.1) | 76.1 (76.0, 76.1) | 76.1 (76.0, 76.2) | 76.1 (76.0, 76.2) | 76.1 (76.0, 76.2) |

Model A is the final main model (age, age2, education, smoking, province) plus health behaviours (alcohol, diet, physical activity and sleep)

Model B is the final main model (age, age2, education, smoking, province) plus psychological factors

Model C is the final main model (age, age2, education, smoking, province) plus marital status

Model D is the final main model (age, age2, education, smoking, province) plus chronic conditions

Model E is the final main model (age, age2, education, smoking, province) plus anthropometry (body mass index, waist circumference).
