# Supplementary material for: Cardiovascular risk in aging adults with double deficits in social support: A gender-sensitive, cross-sectional analysis of the CLSA cohort
Source: PLoS One. 2025 Jun 20;20(6):e0309634. doi: 10.1371/journal.pone.0309634 (PMC12180724; doi:10.1371/journal.pone.0309634)
Supplement: Attachment — Submitted filename: Plos One_Response to Reviewers_dec 2.docx [file pone.0309634.s003.docx]

We thank both reviewers for their feedback on this manuscript. We have addressed each comment point-by-point below and we have taken the opportunity to edit the manuscript to reduce the overall word count.

**Reviewer #1:**

Title: OK.

Abstract:

According to the expressed objectives, the method of exposure measurements related to social supports are not addressed in the method part of this section.

The concern of the reviewer and what needs amendment is unclear to us. We stated in the Methods that the social support measures were self-reported. Given the word limit, we are constrained in providing the detail that each measure is based on questions taken from the Medical Outcomes Study (MOS) which is explained in the main text. We have revised the Methods in the abstract to read: “Cross-sectional study of 28,779 adults (45-85 years) in the Canadian Longitudinal Study on Aging Comprehensive cohort (2012-15) with self-reported measures of informational, tangible, emotional and belonging support, and clinically measured body mass index, waist circumference and blood pressure.”

Please show the results of the study numerically instead of narratively. In this case the conclusion will be sensible for the readers.
Thank you for this suggestion which we have addressed as we now provide numeric data on the key findings among women as described in the Results of the main text.

Keywords: Ok.

Introduction: Please show the novelty of the study in this section. It is also desirable to show the expected practical use of the results of the study in this section.

We appreciate the opportunity to highlight the novelty of this research and its practical implications. We have added new text (underlined) to the Introduction as follows:

*“Thus, it remains an empirical question to determine if one gender is overall more vulnerable to poor CVRFs from double deficits in support, or if women’s and men’s vulnerability varies by which combination is specified. The additive and interactive CVRF effects from different social supports deserve further exploration from a gender lens.*

*This cross-sectional study aimed to fill a knowledge gap on the effects of multiple social supports and their interactions in relation to major CVRFs in an aging population using gender-based analysis. […] If results show that double deficits in social support have synergistic effects on CVRFs and/or gender differences, then public health and social prescribing may need to simultaneously address multiple types of social support or target each gender, or both.”*

Material and method: Please notice to the following issues in this section:
- I think it is necessary to define the sampling method and setting of the main study of population-based Canadian Longitudinal Study on aging in more detail.

We have added some details on the sampling method and setting of the CLSA comprehensive cohort.

- Please state how non-response was managed in this study.

As previously indicated, this cross-sectional study used complete cases for analysis. Although the overall response rate at baseline was low compared to other nationally representative surveys in Canada, the original cohort used a stratified random sampling method and key CLSA measures for the entire cohort are comparable with estimates generated from Canadian census data. Notably, the CLSA has very high retention rate (>97%) suggesting that those who participated in the cohort are highly engaged.

- I think it is essential to define how CVD related risk factors were measured.

As requested, we have added the details from the CLSA standard operating procedures for the instruments used to measure height, weight, waist circumference (the blood pressure instrument was already detailed). Definitions were previously provided in the relevant Methods section.

- Please define the methods used for validating and reliability of the responses used for measuring variables used for assessing the social support. It is also essential to define these variables in detail.

While we appreciate this reviewer’s interest in the details of the MOS scale, the word limit prohibits us from detailing the methods used to validate the MOS instrument and determine its reliability. We have revised the text to explain that: “*The CLSA social support questions were based on the Medical Outcomes Study (MOS) Social Support Survey, which is a validated instrument with good reliability.[17]*” Given the extensive use of the MOS scale and social support questions in the CLSA, we believe readers can obtain more methodological details elsewhere by referencing the CLSA protocol (ref 19) and the original paper on social support (ref 17). We have included information on the summary scores for each social support with the score ranges that were calculated by summing the responses to the relevant question for each type of support.

- The definition of the variables shown under the section of “covariables” shall be defined. For instance, what exactly do you mean by physical activity or weekly alcohol intake?

Due to the word limit, we have place details about the covariables used in sensitivity analysis into a separate Supplementary Methods file. This now explains the following:

“*Chronic disease covariables for robustness checks included binary variables (yes, no (reference)) for hyper- and hypothyroidism; rheumatoid arthritis; asthma; CVD; cancer, osteoporosis; diabetes; Parkinson's; and stroke; and yes/no variable for medications relevant to each chronic condition. Marital status covariable had 4 categories (married / living as married (reference group), never married, widowed, divorced/separated) based on 6 original response options questions (married, living as married, never married, widowed, divorced and separated). Multiple covariables of lifestyle factors were included in robustness checks. Alcohol consumption frequency was dichotomized as weekly or more (every day, 4-5 times a week, 2-3 times a week, once a week) versus the reference group of less than weekly (2-3 times a month, about once a month, less than once a month or never) based on responses to the CLSA question “about how often during the past 12 months did you drink alcohol?” Sleep duration (hours) was based on the self-reported average number of hours of actual sleep CLSA participants had per night in the past month and sleep quality was defined as very satisfied or satisfied (reference) versus neutral, dissatisfied or very dissatisfied in response to the CLSA question “how satisfied or dissatisfied are you with your current sleep pattern?”. The amount of daily physical activity was measured as a Physical Activity Scale for the Elderly (PASE) score that was calculated from reported frequencies and durations of participation in 12 different activities over the previous week and then converted into hours-per-day values. Total times per day of combined fruits and vegetables was assessed using dietary data collected by CLSA’s Short Diet Questionnaire (a 36-iem validated food frequency questionnaire) which first asked whether they consumed fruits and four vegetables daily, weekly, monthly or yearly followed by the question ‘how many times’ for each frequency response (e.g. twice a day, three times a week, once a month); CLSA converted all SDQ questions into times per day. Three psychological factors were measured by the Center for Epidemiological Studies Depression scale (CESD) (score range 0-30); life close to ideal question from the Life Satisfaction Scale (agree (reference), neither, disagree) and depression medication (yes/no). Biological sex risk factors related to women’s reproductive status were the number of biological children (numeric), menopause status (yes (reference), no) and hormone replacement therapy use (ever (reference), never).*

- Please clarify the variables considered for forming the PCs. It is also essential to show the internal consistency of these variables as a proxy for validating this procedure.

Thank you for your valuable comment. We are happy to clarify the variables considered for forming the principal components (PCs) and explain the internal validation procedure.

The variables included in the principal component regression (PCR) were functional ties and all of their two-way interactions. These variables were chosen due to their high intercorrelations, which raised concerns of multicollinearity. To determine the appropriate number of PCs to retain in the regression model, we employed R-squared and root mean squared error of prediction (RMSEP) criteria. Specifically:

- R-squared: We selected the number of PCs that explained the majority of the variance in the original variables while avoiding overfitting.

- RMSEP: Using a cross-validation approach, we minimized the RMSEP to identify the optimal number of PCs that balanced predictive accuracy and model simplicity.

This process ensured that only PCs contributing meaningfully to the predictive power of the model were retained, while irrelevant or noise-inducing PCs were excluded. Together, these criteria serve as an internal validation, demonstrating that the dimensionality reduction procedure effectively captured relevant information while avoiding overfitting. We have added this explanation to the manuscript to clarify the validation process. The revised text reads:

*"Three main steps are involved in fitting a PCR model: 1) Compute principal components (PCs) on correlated covariates (functional ties and all of their two-way interactions); 2) Run a regression model on PCs and other independent cofounders; and 3) Back transform the coefficients’ estimate of the PCs in the regression model to the coefficient of original correlated predictors by using the PCs’ scores. A subset of PCs will be used in the regression model in Step 2 in order to avoid multicollinearity issues between the correlated covariates. We employed R-squared and root mean squared error of prediction (RMSEP) criteria to decide how many PCs should be dropped from the regression model in Step 2.[24] PCs were retained based on their contribution to variance explained and their ability to minimize RMSEP, ensuring robust model performance without overfitting. In most cases, only the last one or two PCs were removed from the regression models."*

- It seems the models, the results of which are shown in figures 2 and 3 are not addressed clearly in this section.

Thank you for pointing this out. We appreciate the opportunity to clarify. The models underlying Figures 1 and 2 (and similarly for Figures 2 and 3) were fitted using PCR, adjusting for all available covariates. These models were designed to estimate the adjusted means of BMI and WC by levels of social support (low vs. high) separately for men and women. We have now revised the manuscript to explicitly describe the modeling approach in the relevant section to ensure clarity. For example, the revised text now reads:

*"Figures 1 and 2 present the adjusted means (prediction mean derived from the fitted models) for BMI and WC, respectively, stratified by levels of social support (low vs. high) in men and women. These figures illustrate the association between social supports (or its absence) and variations in BMI and WC."*

as well as

*"Similarly, Figures 3 and 4 present the adjusted means (prediction mean derived from the fitted models) for SBP and DBP, respectively, stratified by levels of social support and gender."*

- According to the above-mentioned issues the following section of result and discussion are not justifiable.

We have responded to the above issues to provide greater clarity. We respectfully disagree and believe that our results and discussion are indeed well justified given the methods we employed are sound for the research question and the highly correlated nature of data on the exposures of interest.

**Reviewer #2:**

• A recent systematic review (as following) examined the effect of social support on cardiovascular risk. What was the research gap for the current study?
- Singh M, Nag A, Gupta L, Thomas J, Ravichandran R, Panjiyar BK. Impact of Social Support on Cardiovascular Risk Prediction Models: A Systematic Review. Cureus. 2023 Sep 24;15(9):e45836. doi: 10.7759/cureus.45836. PMID: 37881384; PMCID: PMC10597590.

Thank you for noting this article publication of a review article that examined social support (undefined) with respect to risk prediction models of cardiovascular events or outcomes in the entire adult population (19+ years). We note that there are a number of methodological flaws to this publication not least being a poor search strategy (PubMed and Medline are basically the same, and Google Scholar is not actually a replicable bibliometric database). This publication only included clinical trials, RCTs and other reviews but did not reflect population-based observational evidence which is relevant to public health and the current study. The current research is quite distinct from this review in several ways: 1) we distinguish and explicitly examine 4 different types of social support; 2) we consider the additive and interactive effects of combinations of social support; 3) we focus on risk factors of CVD (and not risk prediction models of CVD events or sequelae such as hospitalisation); 4) we apply a gender lens to unpack the potential differential in associations for women and men; and 5) we focus on the later life transition of middle-age and older adults as a uniquely vulnerable group and relevant to healthy aging strategies.

• Among multiple risk factors of cardiovascular diseases, only two anthropometric measures (body mass index and waist circumference) in addition to systolic and diastolic blood pressure have been considered as the indicators of cardiovascular disease (CVD) risk. Some other important factors such as physical activity, nutrition pattern, blood glucose and lipid profile were not considered. They should be mentioned in limitations of the study. Also, these factors might change the association between social support and cardiovascular risk.
We agree with the reviewer that there are a number of recognised physiological (and behavioural) risk factors of CVD and these could change the association between social support and cardiovascular risk. We wish to clarify our rationale for the deliberate focus on obesity and hypertension which are strong physiological determinants of aging since the context of this study is an understanding of the social dimension of aging from a gender perspective. Both obesity and hypertension are not only the two most significant physiological risk factors of CVD but are particularly important for healthy aging from a health equity perspective. Obesity is most prevalent among older age adults and is a major risk factor not only for hyperglycemia and diabetes, high cholesterol and atherosclerosis but also for hypertension and heart disease. Worldwide adult obesity has more than doubled since 1990 and extreme obesity is accelerating fastest among females in Canada. Notably, it has been shown that overweight and obesity are risk factors of MI and IHD regardless of the presence of metabolic syndrome which includes dyslipidemia, high blood glucose and high blood pressure suggesting that metabolic syndrome is no more valuable than BMI in identifying individuals at risk of CVD events (Thomsen and Nordestgaard. *JAMA Intern Med*. 2014;174(1):15-22). From a gender perspective, it is important to note that excess waist circumference is a key component of metabolic syndrome which leads to higher risk for CVD, mortality and stroke among women (RR 2.9, 2.6 and 2.6) than men (2.1, 1.96 and 2.0). Similarly, hypertension is also one of the most prevalent modifiable CVD risk factors for both women and men, but significantly increases in severity with age for women and is more prevalent in post-menopause compared to age matched men. More importantly, women with hypertension are less likely to be diagnosed than men and hypertension is less well controlled among women, even among those taking antihypertensive treatments.

We did in fact consider behavioural risk factors (i.e. physical activity, sleep, alcohol, FV intake) as well as other physiological factors (i.e. diabetes) that were noted by the reviewer which we accounted for as additional covariables in sensitivity analysis. We have provided more detail on the physical activity variable, the healthy eating variable and the chronic condition variable (diabetes) in the Supplemental Methods. Moreover, we note that we had previously stated that our results for the main associations were robust to these other factors as potential confounders. Indeed, this extensive investigation demonstrates the strength of our work. We also acknowledged that we could not consider blood sample data as this was not available to us at the time of data request (this was noted in our limitations section).

To address any lack of clarity for readers, we have restructured the first paragraph of the introduction to open with healthy aging as the context of this work and added the specific variables in Supplementary Methods and highlighted the inclusion of behaviours to our paragraph on study strengths.

• In “Results” of “Abstract” section, it is recommended to provide more statistical findings.

In line with a similar comment from Reviewer 1, we have added numerical data to the abstract.

• “Keywords” are recommended to initiate with capital letters.

We have amended the Keywords based on the relevant Medical Subject Heading terms.

• Why did the authors initiate the phrase of “Comprehensive cohort” with a capital letter in “Methods” section?

The CLSA is comprised of the Tracking Cohort and the Comprehensive Cohort which are formal names and thus capitalised as titles.

• Please provide a brief description of the four types of social support in “Introduction” section.

Due to the journal’s word limit, there is not scope to add a description of each type of support in the Introduction and this information is already provided in the Methods. However, we have revised a former sentence to more explicitly identify the four types of social support that are relevant to CVD risk factors from a gender perspective: *“In addition, four types of social support (tangible, belonging, informational and emotional) were independently associated with adiposity among women but not men [16]”*

• Please check the sentences in Lines 170-175. The verbs should be written in past tense.

Thank you, we have corrected to past tense.
