# Supplementary material for: Cardiovascular risk in aging adults with double deficits in social support: A gender-sensitive, cross-sectional analysis of the CLSA cohort
Source: PLoS One. 2025 Jun 20;20(6):e0309634. doi: 10.1371/journal.pone.0309634 (PMC12180724; doi:10.1371/journal.pone.0309634)
Supplement: Attachment — Submitted filename: Plos One_2nd Response to Reviewer_feb 10.docx [file pone.0309634.s004.docx]

We thank Reviewer 2 for accepting that we have adequately addressed all the Reviewer’s previous comments.

**Reviewer #1:**

Reviewer #1: this is a very important study in terms of the subject. however please notice to teh following comments: We appreciate Reviewer 1’s recognition of the importance of the topic of social support and physiological determinants of aging in women and men.

Title: according to the proposed objective it is better to convey the title with a SMART format (e.g. “the association of social support with cardiovascular disease risk factors in middle age adults”). We understand there are many ways to construct a title, and the SMART format only requires that there be a specific title that is clear, concise and directly reflects the topic of the paper. We believe the current title as is aligns with the SMART objective of conveying the key aspects of the paper and make it easy to understand the subject matter and context at a glance. That is, the title as it is written contains specific information about the study design, the unique focus on gender and on the interactive association of social supports as well as the data source (CLSA) is specified as required by the CLSA data access agreement. We defer to the Editor on this change given that Reviewer 2 indicated the title was ok.

Abstract: Please define the proposed dependent and independent variables explicitly in the material and method part of this section. On the other hand, please show the effect measures of the association of social support with the expressed risk factors. In this case the conclusion will be more sensible for the readers. We have clarified the independent and dependent variables in the Abstract methods and revised the final sentence to include the linear and logistic regression with PCR for interactive association: “*We used stratified multivariable linear and logistic regression with principal component regression for interactive associations to post-estimate adjusted means and 95% CIs”.* Other edits were made so the abstract would be within the 300 words.

Keywords: OK

Introduction: I think it is necessary to express the novelty of the study explicitly in this section. Thank you for this suggestion, we have revised the last paragraph of the Introduction to explicitly express the study’s novelty: “*This cross-sectional study is novel in addressing a knowledge gap on additive and interactive CVRF effects from four different social supports and on potential differences by gender.*”

Material and method: Please notice to the following issues in this section:
- Please define the main study of CLSA in more detail (especially in terms of sampling method and measurement tools and reliability and validity of the variable measurements). Although we previously included information on the sampling method, physical measurement tools and the valid and reliable social support instrument, we nevertheless give more details in an expanded Supplementary Methods file. However, it goes beyond the scope of this paper to further define the main CLSA cohort in terms of the reliability and validity of all the variable measurements as these are quite extensive and not applicable to the current paper. Readers are encouraged to refer to the two publications on the CLSA cohort and the CLSA website which contains all the documentation on all the CLSA measurement tools. We trust that the additional detail on the main CLSA study related to the variables we used, is now satisfactory for the Reviewer.

- In contrast to detailed description of the social support categories, it is not clear how different types have been measured. We are unsure what exactly the Reviewer seeks us to amend, we have provided additional detail on the social support variables in the Supplementary Methods, including a Table S1 with the four support types, the exact question item from the CLSA SSA module and the scoring approach. We also amended the main text to provide the coding for each of the five response options to each of the 19 social support questions. We added three new references to support the classification of four types of social support that departs from the MOS subscales since one subscale combines both emotional and informational support together.

- The method of additive and multiplicative association assessment shall be defined in more detail. On the other hand, it is necessary to principal component analysis in more detail so that it will be clarified which variables have been considered as the recruited assets of PCA. We have prepared two tables (for continuous and binary outcomes) containing the number of PCs used for each model (by outcome and gender). And, for additional specificity, we created a third table containing the PCA scores for only a single model as an example (continuous BMI in males). This table shows that all the functional ties along with their interactions showed up in every single PC, and therefore, no matter how many PCs we selected for each model, all the ties along with their interactions were included in all of our models. In addition, we have revised the text in the Statistical Analysis section to elaborate on how exactly we fitted the PCR models. This includes a sentence to refer readers to the newly generated tables containing how many PCs we used for each PCR (now in Supplementary Material).

- Please define the used sensitivity analysis in more detail. We have amended the sentence to further explicate that: “*Sensitivity analyses were used to test the robustness of the interactive associations to additional confounding in separate models further conditioned on chronic disease, marital status, health behaviours, psychological factors and reproductive status (covariable details in Supplementary Methods)*.”

- It is necessary to express how different variables were considered to be recruited for the multivariable analysis. We included the fact that our variable selection for adjustment was based on the literature, known risk factors, biological plausibility and specialized content knowledge.

Results: part of this section is not justifiable according to the above-mentioned issues. The above issues have been addressed.

Discussion: Please express the limitations of the study in this section as well. We believe we have sufficiently expressed the limitations of the study as it is currently written and we are unclear from what precise actions we need to take to respond to this general comment. We defer to the Editor on this matter.

References: OK
