# Supplementary material for: Cardiovascular risk in aging adults with double deficits in social support: A gender-sensitive, cross-sectional analysis of the CLSA cohort
Source: PLoS One. 2025 Jun 20;20(6):e0309634. doi: 10.1371/journal.pone.0309634 (PMC12180724; doi:10.1371/journal.pone.0309634)
Supplement: Attachment — Submitted filename: Reviewer Comments to Author_R3.docx [file pone.0309634.s005.docx]

Reviewer Comments to Author

Reviewer #1: thanks to distinguished authors, I think most of the comments are addressed. Please just notice to the following minor issues:

We appreciate the careful review and insights previously offered by the reviewer to improve our paper. We are delighted that our revisions are well received.

- I could not understand why just findings related to women are shown in the abstract.

We wish to clarify that the abstract mostly reports findings for women because it was only in women that we observed that combinations of dual deficits in social support were associated with CVRFs to a greater extent than the combinations of social support where only one support was in deficit. By contrast, there was no clear pattern of this additive joint association observed among men or the figures showed a flat pattern of mean CVRFs levels across the combinations of support. We also focus on results for women given the paucity of CVD research that provides a gender lens or reports disaggregated data.

- - The supplementary material was not included.
We are uncertain why the reviewer did not receive the Supplementary Material. Our records show within the online system that the Supplementary Methods were uploaded on February 10, 2025 at 8:46pm, along with our second Response to Reviewers. We have now included this file within the main manuscript file to ensure that the Reviewer sees this information without a glitch in the system.

- Please the reliability indices used for the assessment of the responses social support variables.
We have included specific reliability indices in the Methods, with a new citation, as follows: “which has overall excellent internal reliability (Cronbach’s alpha > 0.90) and good test-retest reliability (ICC ≥ 0.84) [16] and good reliability of each dimension (CFA > 0.90).[23]”

- I think it is necessary to define the method of interaction (both additive and multiplicative ones) in more detail. (This is issue may be due to the fact that supplemental material was not included).

We have defined in the Methods that the method of interaction was the use of a cross-product term between each pair of social supports. This specificity has been highlighted and reinforced for the relevant text in the Methods. To clarify, we did not quantify the interaction on additive or multiplicative scale but rather interpreted our findings through visual inspection of the graphs and the mean values of CVRFs for each combination of the four different social supports. Thus, we have amended the manuscript text to make this approach more clear.
